# Connectomics-based resting-state functional network alterations predict suicidality in major depressive disorder

**DOI:** 10.1038/s41398-023-02655-4

**Published:** 2023-11-27

**Authors:** Qing Wang, Cancan He, Zan Wang, Dandan Fan, Zhijun Zhang, Chunming Xie, on behalf of REST-meta-MDD Consortium, Chao-Gan Yan, Xiao Chen, Le Li, Francisco Xavier Castellanos, Tong-Jian Bai, Qi-Jing Bo, Guan-Mao Chen, Ning-Xuan Chen, Wei Chen, Chang Cheng, Yu-Qi Cheng, Xi-Long Cui, Jia Duan, Yi-Ru Fang, Qi-Yong Gong, Wen-Bin Guo, Zheng-Hua Hou, Lan Hu, Li Kuang, Feng Li, Kai-Ming Li, Tao Li, Yan-Song Liu, Zhe-Ning Liu, Yi-Cheng Long, Qing-Hua Luo, Hua-Qing Meng, Dai-Hui Peng, Hai-Tang Qiu, Jiang Qiu, Yue-Di Shen, Yu-Shu Shi, Chuan-Yue Wang, Fei Wang, Kai Wang, Li Wang, Xiang Wang, Ying Wang, Xiao-Ping Wu, Xin-Ran Wu, Guang-Rong Xie, Hai-Yan Xie, Peng Xie, Xiu-Feng Xu, Hong Yang, Jian Yang, Jia-Shu Yao, Shu-Qiao Yao, Ying-Ying Yin, Yong-Gui Yuan, Ai-Xia Zhang, Hong Zhang, Ke-Rang Zhang, Lei Zhang, Ru-Bai Zhou, Yi-Ting Zhou, Jun-Juan Zhu, Chao-Jie Zou, Tian-Mei Si, Xi-Nian Zuo, Jing-Ping Zhao, Yu-Feng Zang

**Affiliations:** 1 Department of Neurology, Affiliated ZhongDa Hospital, School of Medicine, Southeast University, Nanjing, Jiangsu 210009 China; 2 Institute of Neuropsychiatry, Affiliated ZhongDa Hospital, Southeast University, Nanjing, Jiangsu 210009 China; 3 The Key Laboratory of Developmental Genes and Human Disease, Southeast University, Nanjing, Jiangsu 210009 China; 4 Key Laboratory of Behavioral Science, Institute of Psychology, Chinese Academy of Sciences, Beijing, 100101 China; 5 Department of Psychology, University of Chinese Academy of Sciences, Beijing, 100049 China; 6 Department of Child and Adolescent Psychiatry, New York University School of Medicine, New York, NY10016 US; 7 Nathan Kline Institute for Psychiatric Research, Orangeburg, NY 10962 USA; 8 Anhui Medical University, Anhui, 230022 China; 9 Beijing Anding Hospital, Capital Medical University, Beijing, 100054 China; 10 The First Affiliated Hospital of Jinan University, Guangzhou, Guangdong 510630 China; 11 Department of Psychiatry, Sir Run Run Shaw Hospital, Zhejiang University School of Medicine, Hangzhou, Zhejiang 310016 China; 12 Department of Psychiatry, The Second Xiangya Hospital of Central South University, Changsha, Hunan 410011 China; 13 First Affiliated Hospital of Kunming Medical University, Kunming, Yunnan 650032 China; 14 Department of Psychiatry, First Affiliated Hospital, China Medical University, Shenyang, Liaoning 110001 China; 15 Department of Psychiatry, Shanghai Jiao Tong University School of Medicine, Shanghai, 200240 China; 16 Department of Radiology, Huaxi MR Research Center, West China Hospital of Sichuan University, Chengdu, Sichuan 610041 China; 17 Psychoradiology Research Unit of Chinese Academy of Medical Sciences, West China Hospital of Sichuan University, Chengdu, Sichuan 610041 China; 18 Department of Psychosomatics and Psychiatry, Zhongda Hospital, School of Medicine, Southeast University, Nanjing, Jiangsu 210096 China; 19 Department of Psychiatry, The First Affiliated Hospital of Chongqing Medical University, Chongqing, 400016 China; 20 Mental Health Center, West China Hospital, Sichuan University, Chengdu, Sichuan 610041 China; 21 Department of Clinical Psychology, Suzhou Psychiatric Hospital, The Affiliated Guangji Hospital of Soochow University, Suzhou, Jiangsu 215137 China; 22 Faculty of Psychology, Southwest University, Chongqing, 400716 China; 23 Department of Radiology, The First Affiliated Hospital, College of Medicine, Zhejiang University, Hangzhou, Zhejiang 310058 China; 24 Xi’an Central Hospital, Xi’an, Shaanxi 710003 China; 25 National Clinical Research Center for Mental Disorders, Peking University Sixth Hospital, Beijing, 100191 China; 26 Key Laboratory of Mental Health, Ministry of Health, Peking University, Beijing, 100191 China; 27 Department of Psychiatry, The Fourth Affiliated Hospital, College of Medicine, Zhejiang University, Hangzhou, Zhejiang 310058 China; 28 Institute of Neuroscience, Chongqing Medical University, Chongqing, China; 29 Chongqing Key Laboratory of Neurobiology, Chongqing, 400016 China; 30 Department of Neurology, The First Affiliated Hospital of Chongqing Medical University, Chongqing, 400016 China; 31 The First Affiliated Hospital of Xi’an Jiaotong University, Shanxi, 710061 China; 32 First Hospital of Shanxi Medical University, Taiyuan, Shanxi 030001 China; 33 Center for Cognition and Brain Disorders, Institutes of Psychological Sciences, Hangzhou Normal University, Hangzhou, Zhejiang 311121 China; 34 Zhejiang Key Laboratory for Research in Assessment of Cognitive Impairments, Hangzhou, Zhejiang 311121 China

**Keywords:** Depression, Diagnostic markers

## Abstract

Suicidal behavior is a major concern for patients who suffer from major depressive disorder (MDD). However, dynamic alterations and dysfunction of resting-state networks (RSNs) in MDD patients with suicidality have remained unclear. Thus, we investigated whether subjects with different severity of suicidal ideation and suicidal behavior may have different disturbances in brain RSNs and whether these changes could be used as the diagnostic biomarkers to discriminate MDD with or without suicidal ideation and suicidal behavior. Then a multicenter, cross-sectional study of 528 MDD patients with or without suicidality and 998 healthy controls was performed. We defined the probability of dying by the suicide of the suicidality components as a ‘suicidality gradient’. We constructed ten RSNs, including default mode (DMN), subcortical (SUB), ventral attention (VAN), and visual network (VIS). The network connections of RSNs were analyzed among MDD patients with different suicidality gradients and healthy controls using ANCOVA, chi-squared tests, and network-based statistical analysis. And support vector machine (SVM) model was designed to distinguish patients with mild-to-severe suicidal ideation, and suicidal behavior. We found the following abnormalities with increasing suicidality gradient in MDD patients: within-network connectivity values initially increased and then decreased, and one-versus-other network values decreased first and then increased. Besides, within- and between-network connectivity values of the various suicidality gradients are mainly negatively correlated with HAMD anxiety and positively correlated with weight. We found that VIS and DMN-VIS values were affected by age (*p* < 0.05), cingulo-opercular network, and SUB-VAN values were statistically influenced by sex (*p* < 0.05). Furthermore, the SVM model could distinguish MDD patients with different suicidality gradients (AUC range, 0.73–0.99). In conclusion, we have identified that disrupted brain connections were present in MDD patients with different suicidality gradient. These findings provided useful information about the pathophysiological mechanisms of MDD patients with suicidality.

## Introduction

Major depressive disorder (MDD) is a common psychiatric disorder characterized by an inability to experience pleasure/reward (anhedonia) [1], affecting nearly 350 million people worldwide [2]. MDD is a major risk factor for suicide, with 7% of men and 4% of women with MDD who die due to suicide every year [3]. It has been reported that suicide occurs in a three-step gradual process consisting of suicidal ideations (SIs), suicidal attempts, and suicidal death [4–6]. Approximately 23% of those who have committed suicide had previously attempted it [7]. Therefore, it is important for suicide prevention to identify MDD patients with SI and suicidal attempts and study their underlying mechanisms. However, the precise molecular mechanisms associated with suicidality are poorly understood. In addition, it is a difficult task to assess suicidality in patients with MDD due to the subjectivity of psychological scales and the unwillingness of patients to disclose their thinking or the acts they have committed [8–10]. Thus, it is urgent to find an objective biomarker to identify suicidality in MDD patients and to understand of neural circuits underlying the pathology of suicidality.

Neuroimaging and behavioral studies have recently centered on network-based structural and functional alterations of individuals at risk of suicide. Previous studies postulated that fronto-limbic system is the central circuit underlying the suicidal process under depressive conditions [11–15]. MDD patients with SI showed reduced intrinsic functional connectivity (FC) between the rostral anterior cingulate cortex and the right middle temporal pole, in comparison with MDD patients without SI and healthy controls (HCs), and these impairments in connectivity would be positively correlated with SI severity [13]. It has also been found that MDD patients with suicidal attempts had significantly higher FC strength in the bilateral dorsomedial prefrontal cortex and the right orbitofrontal cortex than patients without suicidal attempts, which is thought to be associated with a higher risk of suicidal behavior (SB) in MDD patients [16]. Meanwhile, numerous cross-sectional neuroimaging studies report decreased cortical gray matter and disturbed frontal-subcortical white matter integrity in MDD patients with SI and MDD patients with SB compared with HCs [17–19]_._ Furthermore, previous studies aver that cortical thickness of ten regions within fronto-temporal-parietal system act as top-ranked classifiers that could differentiate suicide attempts from SI in MDD patients [20]. In addition, neural representations of suicidal and emotional concepts with a machine learning approach in suicidal youth could classify youth with or without suicide [21]. These findings initially revealed that frontal-limbic system contributed substantially to suicidality in MDD patients. Populations with different elements of suicidality have different probabilities of eventually dying by suicide. Here, we defined the probability of dying by suicide of the suicidality components as a ‘suicidality gradient’. And the suicidality gradient of MDD patients without SI is the smallest and the gradient of MDD patients with SB is the largest. However, there is a rarity of data on how individual resting-state networks (RSNs) change dynamically and how functionally distinct networks interact with each other as the suicidality gradient increases in MDD patients.

Based on prior studies, we hypothesized that suicidal gradient in MDD patients might arise from disturbances in macroscale brain RSNs and altered brain connections may represent powerful diagnostic biomarkers to discriminate MDD with or without SI and SB. First, the current study mapped the dynamic trajectory of large-scale RSNs roles and their clinical significance with dynamic network-based analysis in MDD patients with suicidality gradient. Second, common and specific network connections associated with different suicidal gradients were identified in MDD patients. Third, the support vector machine (SVM) model was used to explore the role of these abnormal neuroimaging characteristics as objective diagnostic biomarkers in classifying MDD patients with different suicidal gradients. Depression shows gender specificity in which the incidence rate of MDD is approximately twofold higher in women than in men [22]. Therefore, the current study finally also explored the potential effects of age and gender on large-scale suicide-related networks. These findings were also reproducible across included sites in an independent validation dataset.

## Methods

### Study participants

A total of 528 MDD patients and 998 HCs were recruited from the REST-meta-MDD consortium [23] and the Department of Psychiatry at Henan Provincial Mental Hospital. All included subjects were 18–65 years of age, with at least 5 years of education. All patients met the Diagnostic and Statistical Manual of Mental Disorders IV criteria for MDD [24], and had a total score ≥8 on the 17-item Hamilton Depression Rating Scale (HAMD)[25] at the time of scanning. *Among the previously mentioned MDD patients, 169 individuals had a medication history; nevertheless, they had refrained from taking medication for a duration of at least three weeks at the time of enrollment.* Based on a 17-item HAMD suicide item score, MDD patients were categorized into five categories: a score of 0 was defined as MDD without suicidal ideation (MDDNSI); score of +1 was defined as MDD with mild suicidal ideation (MDDmSI); score of +2 was defined as MDD with moderate suicidal ideation (MDDmoSI); score of +3 was defined as MDD with severe suicidal ideation (MDDSSI); and score of +4 was defined as MDD with SB (MDDSB). HCs were randomly divided into HC and verification groups. Detailed information on all subjects is shown in Table 1, Supplementary Table 2, and Supplementary Table 3. Unless otherwise noted, methods of analysis are described in Supplemental materials.

**Table 1:** Demographic and Clinical Characteristics for All Subjects.

Groups	HC (*n* = 499)	MDD					*F*/*x*^2^ values	*P* Values
		MDDNSI (*n* = 134)	MDDmSI (*n* = 150)	MDDmoSI (*n* = 110)	MDDSSI (*n* = 93)	MDDSB (*n* = 41)		
Age (years)	33.05 ± 12.57	33.78 ± 11.68	34.6 ± 11.48	32.88 ± 11.06	31.41 ± 9.94	28.33 ± 11.70	1.54	0.17
Sex (%, female)	297 (55.83%)	77 (55.40%)	96 (63.15%)	81 (71.68%)^a,d^	67 (67.68%)^b^	23 (53.49%)^i^	16.54	0.006^*^
Education (years)	13.91 ± 3.45	11.79 ± 3.40	11.87 ± 3.72	12.11 ± 3.52	12.38 ± 3.33	11.23 ± 3.39	0.41	0.840
HAMD	n.a.	16.25 ± 7.40	21.84 ± 4.51^c^	23.80 ± 5.77^d^	25.93 ± 6.08^e,g^	27.05 ± 7.28^f,h^	47.52	<0.0001
HAMD-Suicide	n.a.	0 ± 0	1 ± 0	2 ± 0	3 ± 0	4 ± 0	7.65E +15	<0.0001
HAMD-Anxiety	n.a.	4.83 ± 2.62	5.89 ± 2.09^c^	5.73 ± 2.55^d^	6.13 ± 2.53^e^	5.95 ± 2.73	5.32	0.0003
HAMD-Weight	n.a.	0.4 2 ± 0.63	0.58 ± 0.74	0.66 ± 0.79	0.73 ± 0.89^e,g^	1.20 ± 1.01^f,h, i^	6.26	<0.0001
HAMD-Retardation	n.a.	5.52 ± 2.72	7.05 ± 2.01^c^	7.64 ± 2.28^d^	8 .00 ± 2.25^e^	8.15 ± 1.81^f,g^	22.17	<0.0001
HAMD-Sleep	n.a.	3.20 ± 2.04	3.92 ± 1.61^c^	4.10 ± 1.70^d^	4.16 ± 1.71^e^	3.90 ± 2.38	5.78	0.0001
Total disease duration (months)	n.a.	47.4 ± 78.2	38.64 ± 67.42	32.48 ± 57.47	23.06 ± 35.2^e^	40.79 ± 56.01	2.18	0.07
First-episode (yes/no, percent)	n.a.	49.6/50.4	55.9/44.1	64/36	57.6/42.4	46.2/53.8	7.95	0.094

Notes: ^*^p value was obtained by chi-square test; other *p* values were obtained by analyses of variance (ANOVA) among groups. Unless indicated, data are presented as the mean ± standard deviation. Post hoc analyses were used with least significance difference (LSD) correction (*p* < 0.05). All abbreviations can be found in Supplementary Table 8 in the Supplement.

^a^Statistical difference was detected between HC group and MDDmoSI group.

^b^Statistical difference was detected between HC group and MDDSSI group.

^c^Statistical difference was detected between MDDNSI group and MDDmSI group.

^d^Statistical difference was detected between MDDNSI group and MDDmoSI group.

^e^Statistical difference was detected between MDDNSI group and MDDSSI group.

^f^Statistical difference was detected between MDDNSI group and MDDSB group.

^g^Statistical difference was detected between MDDmSI group and MDDSSI group.

^h^Statistical difference was detected between MDDmSI group and MDDSB group.

^i^Statistical difference was detected between MDDmSI group and MDDSB group.

All study sites obtained approval from their local institutional review boards and ethics committees. Also, these research protocols were approved by the Ethics Committee of Henan Provincial Mental Hospital Affiliated with Xinxiang Medical University (approval ID: 2017–08). All participants, their legal guardians, or their legally authorized representatives provided informed consent prior to their involvement in the study.

### Image processing

Scan acquisition was completed within 1 week of assessments. Resting-state fMRI and structural T1-weighted MRI brain scans were acquired at each of the 24 participating study sites (see STable 1 for key data acquisition parameters) and were preprocessed using DPARSF software [26] using a standardized protocol [23]. Briefly, the procedure involved the removal of the first 10 volumes for signal equilibrium, slice-timing correction, head motion realignment, brain tissue segmentation, spatial normalization, and temporal filtering (0.01–0.10 Hz). Friston-24 head motion parameters, liner trends, signals from white matter, cerebrospinal fluid, and whole brain were regressed out from images to control for head motion and physiological noises [27–29]. Subjects with poor image quality or excessive head motion (mean framewise displacement (FD) [30] > 0.2 mm) were excluded from the analysis. Further details are shown in Supplementary materials. After preprocessing, time series for Power 264 functional ROIs were extracted.

### Construction of functional networks

A power atlas [31] was used to partition the brain of each participant into 226 cortical and subcortical areas. Pearson correlation was used to estimate FC between all pairs of regions of interest across all subjects. The study site was added to the covariable file for corrected potential differences in MRI assessment. Network connectivity was subsequently computed within 10 RSNs as defined by previous fMRI studies [31, 32]. *These networks comprise the auditory network (AUD), the cingulo-opercular network (CON), the dorsal and ventral attention network (DAN and VAN), the default mode network (DMN), the fronto-parietal network (FPN), the salience network (SAN), the sensorimotor network (SMN), the subcortical network (SUB), and the visual network (VIS)*. Network connectivity between all pairs of 10 RSNs, as well as between each RSN and all other RSNs (i.e., one-versus-all-others) were computed.

### Statistical analyses

#### Group comparisons of demographic characteristics and network metrics

Group comparisons of demographic characteristics and network metrics across MDD subgroups were undertaken using analysis of covariance (ANCOVA) and significance levels were set at *p* < 0.05 for all tests. In addition, a one-way analysis of variance (ANOVA) test was used to analyze continuous variables, with post hoc least significance difference (LSD) tests for pair-wise comparisons. Chi-squared tests were also used for categorical variables. Notably, each network metric (for instance within-, one-versus-all-others-, and pairwise between-network connectivity (BNC)) was compared across groups using generalized linear model (GLM) analysis adjusted for age, gender, education, and study site as covariables. All p values were adjusted for multiple comparisons (10 within-network metrics + 10 one-versus-all-others-network metrics + 45 pairwise between-network metrics = 65 comparisons) using FDR correction.

### Network-based statistical analysis

The current study first generated a 226 * 226 connectivity matrix for each subject. Network-based statistical analysis (NBS) method was then used to identify common and differential connections of networks between healthy control (HC) and disease groups, as well as various disease subgroups. Each connection identified by NBS with Bonferroni correction satisfied *p* < 0.001.

### Heterogeneity analysis of sex and age

Due to the potential effects of age and sex in dynamic network analysis, Wilcoxon rank sum tests were used to compare the abnormality of network and clinical variables in sex and age. Specifically, all patients were split into younger (age: 18–37) and older (age: 38–65) participants or females and males to obtain sex- and age-related alterations in networks and clinical variables, separately.

### Correlation analysis

The current study computed Pearson’s correlation between network variables and clinical data.

### Machine learning

SVM was used in the current study to classify MDD subgroups and HCs in MATLAB based on a library (LIBSVM) [33]. The current study undertook an overlap analysis of the findings of NBS and established that 31 brain connections showed significant group differences. The links were used in classification using SVM. The data set was randomly split 10 times into training and testing sets. Tenfold cross-validation [34] was applied to the training set to prevent overfitting. The performance of the final machine learning model was quantified by computing accuracy, sensitivity, specificity, and area under the curve (AUC) to reduce the impact of deviations in the distribution of training and testing sets. In addition, the accuracy (ACC) of the testing set was assessed by permutation test with 1000 epochs as described in previous studies [35].

### Validation analyses

All described analyses were repeated using another set of healthy subjects that included 499 subjects to validate whether network role construction could be replicated and whether selected functional connections could be used for classification.

## Results

### Demographic information and clinical performance

Demographic information and characterization of all study subjects are outlined in Table 1. Significant differences in sex, but not age and years of education were observed between MDD subgroup patients and HCs. MDD patients with suicidality showed significantly higher HAMD total scores (*p* < 0.001) and subscales scores including anxiety (*p* = 0.0003), weight (*p* < 0.0001), retardation (*p* < 0.0001), and sleep (*p* = 0.0001), compared with those of MDNSI subjects, indicating that MDD patients R1–2 with suicidality had severer depression compared with MDNSI patients. *Furthermore, there were no statistically significant differences in additional clinical characteristics upon enrollment, including total disease duration and frequency of episodes among the subgroups of individuals with MDD*.

### Network modeling in MDD patients and HC group

**Fig. 1:**
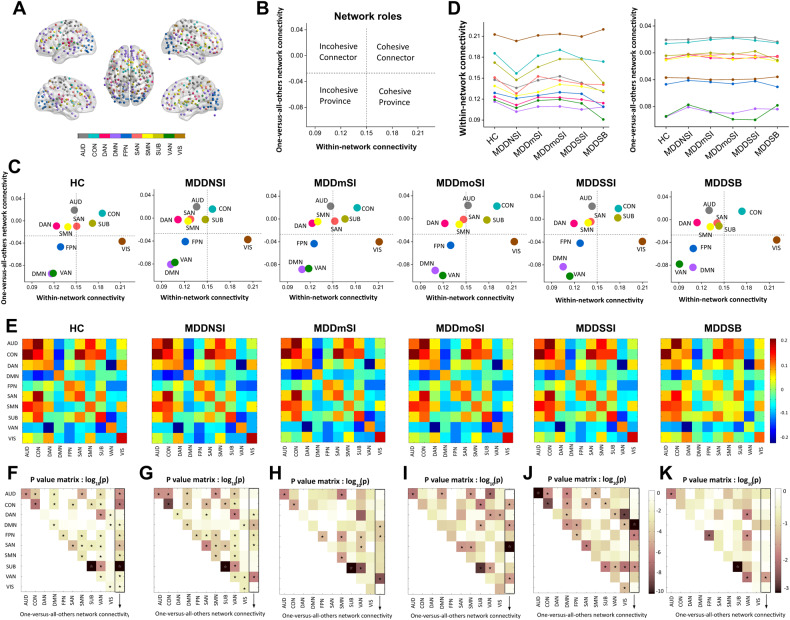
Network roles of five sub-groups of MDD patients and HC group. **A** Node colors represent Power-atlas cortical and subcortical regions consisting of 10 resting-state networks (RSNs). **B**, **C** Network roles in brain networks of HC, MDDNSI, MDDmSI, MDDmoSI, MDDSSI, and MDDSB. **D** Line charts display the dynamic trajectory of within- and one-versus-all-others network connectivity among the five subgroups of MDD patients and the HC group. **E** Within- and pairwise between-network connectivity matrices of five sub-groups of MDD patients and HC group. **F**–**J** *P* value matrix of group differences in within-, one-versus-all-others-, and pairwise between-network connectivity (**F**: HC vs. MDDNSI; **G**: HC vs. MDDmSI; **H**: HC vs. MDDmoSI; **I**: HC vs. MDDSSI; **J**: HC vs. MDDSB; **K**: five MDD sub-groups). The pentacle represents *p* value less than 0.05. All abbreviations can be found in Supplementary Table 8 in the Supplement.

To establish functional links between regionally separated and functionally distinct networks, the current study constructed 10 well-established, large-scale RSNs, which comprised cortical and subcortical regions from Power-atlas [31]: DMN, AUD, CON, DAN, FPN, SAN, SMN, SUB, VAN, and VIS as shown in Fig. 1A. Second, the current study quantitatively measured functional roles of 10 RSNs by mapping group-level, mean within- and between-networks FC, which reflected dynamics of functional synchrony for large-scale networks (Fig. 1B and C). Based on the distribution of mean FC values within- and between networks, these RSNs showed distinctive network roles in suicidality-related MDD patients: 7 RSNs were divided into four network roles including incohesive connector (DAN, SMN), cohesive connector (CON), incohesive province (DMN, VAN, and FPN), and cohesive province (VIS). Other networks (AUD, SAN, and SUB) displayed divergent network roles: AUD and SAN showed dynamic changes between incohesive connector and cohesive connector, whereas SUB displayed a similar pattern (cohesive connector) in MDDSI patients. However, the SUB network displayed the opposite pattern (incohesive connector) in MDDSB and MDDNSI patients, compared with the HC group **(**Fig. 1C). Further, the current study illustrated the dynamic trajectory of averaged FC values within- and between networks, which characterized changes of network links for individuals from HC group to MDDSB patients (Fig. 1D).

### Group-level difference of network connectivity in suicidality-related MDD patients compared to HC group

To quantitatively assess group-level abnormality of natural links between networks, the current study showed network patterns of mean within-network connectivity (WNC) R1–3 and pairwise BNC in HC group and suicidality-related MDD patients (Fig. 1E). *Specific values in* Fig. 1E*can be found in the* Supplementary Table 4. Furthermore, group differences in mean WNC, pairwise BNC, and one-versus-all-others-network were tested (Fig. 1F, K) using the NBS method. Although functional network roles of 7 out of 10 RSNs were stable (DAN and SMN for incohesive connector, CON for cohesive connector, DMN, VAN, and FPN for incohesive province, and VIS for cohesive connector), suicidality-related MDD patients showed significant differences of WNC in SUB, AUD, SAN, and BNC in SMN-AUD and VIS-DAN, whereas AUD, SAN, and SUB also showed differential dynamic network roles from incohesive connector to cohesive connector compared with HC group. In addition, SUB and SAN displayed significant differences in WNC in MDDNSI and MDDSSI patients compared with the HC group, whereas DMN showed consistent differences in mean connectivity of one versus other networks in MDDSB patients compared with the HC group. Notably, VAN was consistently kept in the incohesive province across all subjects, and significant differences in mean FC in one versus other networks in MDD patients with or without SI, but not with SB were observed, compared with the HC group (Fig. 1F and J). In addition, among suicidality-related MDD patients, there were remarkable differences in WNC in SUB, AUD, FPN, and VAN networks, and BNC in the VAN-FPN, VAN-DAN, as well as mean FC values in one versus other networks in VAN (Fig. 1K).

### Mapping abnormal network connections among ten RSNS in suicidality-related MDD patients

**Fig. 2:**
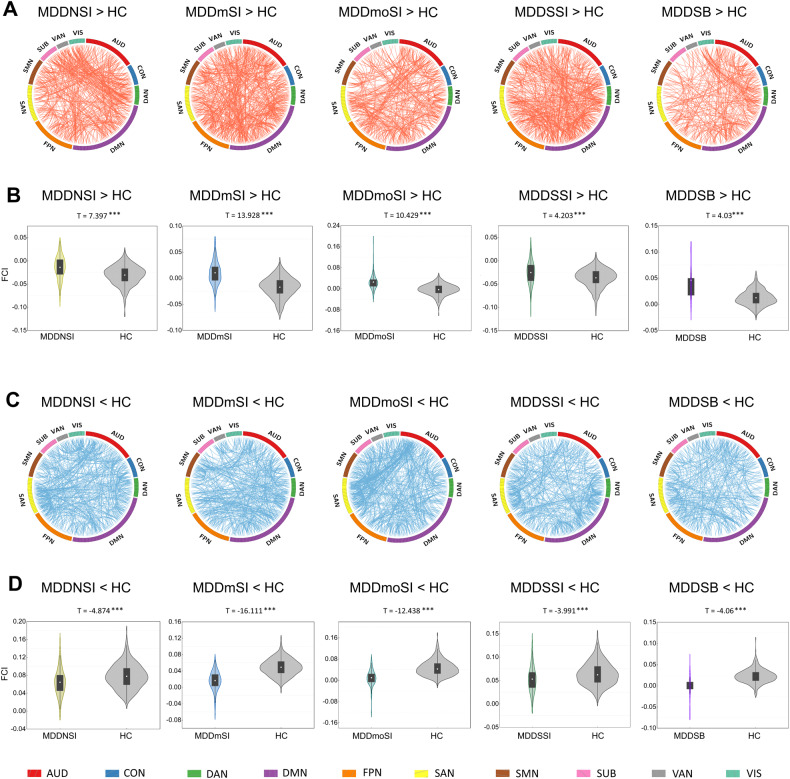
Divergent network connections among the ten RSNs in the five subgroups of MDD patients compared to the HC group. **A**, **C** Significantly increased and decreased network connections among the ten RSNs in the subgroups of MDD patients compared to the HC group. Each square color represents one of the ten networks. Red lines represent increased functional connectivity and blue lines represent decreased functional connectivity. **B**, **D** The violin figures represent the group-level distribution of mean FC from the differential network connections among the ten RSNs in the subgroups of MDD patients compared to the HC group. All abbreviations can be found in Supplementary Table 8 in the Supplement.

To further map abnormal network connections with significant group differences in within-, between- or one versus other networks of ten RSNs in MDD patients, the current study first established increased and decreased network connections in the suicidality-related MDD patients compared with the HC group as shown in Fig. 2A, C. Difference map of regional connectivity strengths in the ten RSNs components was then converted into the composite numerical index to quantitatively assess alterations of WNC and BNC. For example, composite numerical FC was obtained and defined as FC index (FCI) by averaging FC strengths over connections within- and between regions of ten network components. Numerical representation of increased and decreased FCIs are illustrated in MDD subgroup patients relative to the HC group (Fig. 2B, D). Similarly, the current study also identified altered network connections and FCIs in suicidality-related MDD patients compared with MDDNSI patients, or within suicidality-related MDD patients as shown in Supplementary Fig. 3 and Supplementary Fig. 4. Conjunction analysis was then undertaken to obtain overlapping connections, which represented common network connections between arbitrary two groups (Supplementary Fig. 5) [36]. In addition, the current study established that there were 31 pairs of overlapping network connections involved in suicidality-related MDD patients compared with the HC group (Supplementary Table 5). These findings indicate that divergent and convergent brain networks in suicidality-related MDD patients can be detected using large-scale network links.

### Behavioral significance of abnormal network connectivity

**Fig. 3:**
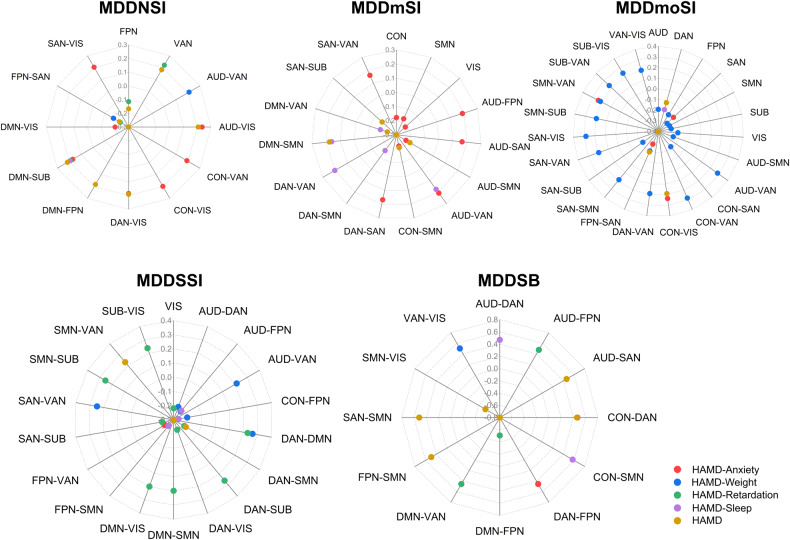
Behavioral correlation between network values and depressive severity in suicidality-related MDD patients. Radar plots showing patterns of association of within- and between-network connectivity with HAMD total scores and subfactors’ scores, including HAMD-Anxiety, HAMD-Weight, HAMD-Retardation, and HAMD-Sleep. All dots in the radar plots represent a statistically significant correlation with Pearson’s correlation coefficients (*p* < 0.05). All abbreviations can be found in Supplementary Table 8 in the Supplement.

To understand the clinical significance of abnormal WNC and BNC in MDD subgroups, the current study conducted Pearson’s correlation between FC strength within- and between networks and clinical variables including HAMD total scores and subfactor scores, including HAMD-Anxiety, HAMD-Weight, HAMD-Retardation, and HAMD-Sleep in MDD patients after controlling for covariables of age, gender, education, and study site. Correlation patterns showed group-level associations of WNC and BNC with depressive severity in MDD subgroup patients and established that distinctive network basis was associated with different symptom dimensions in MDD patients with or without suicidality (Fig. 3). These neural correlation maps directly demonstrated that large-scale brain networks were significantly involved in suicidality gradient-related depression. Detailed information for network basis and correlation values are described in Supplementary Table 6 in Supplementary material.

### Neuroimaging biomarkers for classifying suicidality-related MDD patients

**Fig. 4:**
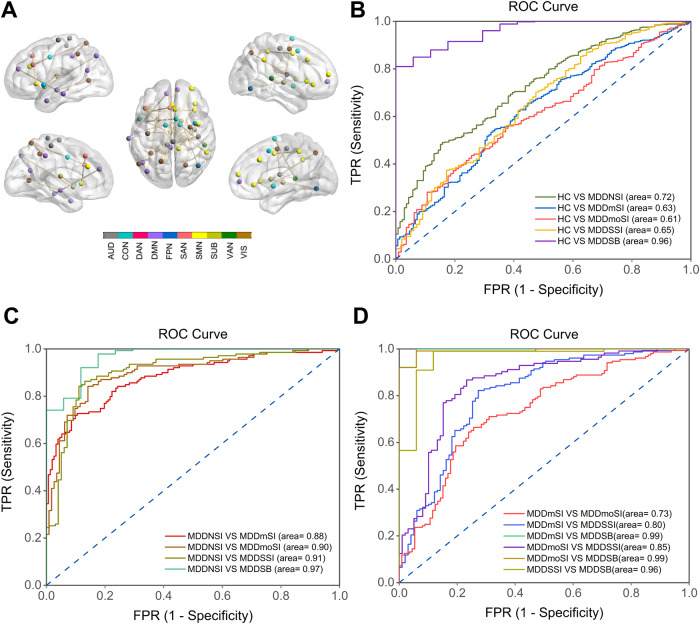
Neuroimaging biomarkers for classifying suicidality-related MDD patients. **A** Thirty-one functional connections were used for the classification between groups. Node colors represent Power-atlas cortical and subcortical regions consisting of ten RSNs. **B**–**D** Functional connections that showed group-level differences were used as the inputs for binary classification (**B**: MDD subgroups vs. NC; **C**: MDDSI subgroups or MDDSB vs. MDDNSI; **D**: among MDDSI subgroups and MDDSB). All *p* values of the area under the curve were <0.001. All abbreviations can be found in Supplementary Table 8 in the Supplement.

The SVM model was used to explore the role of abnormal FC as an objective diagnostic biomarker in MDD patients. Mean FCIs from 31 pairs of network connections were used as input features to the linear support vector classifier (SVC). AUC showed that these FCIs demonstrated a higher capacity to discriminate MDD patients with SB from the HC group (AUC = 0.96). The use of the SVM-trained model as a classifier demonstrated that the SVM-trained model showed better power in separating MDD patients with SI or SB from MDDNSI patients (all AUCs were more than 0.88). Furthermore, FCIs also showed greater potential to discriminate suicidality gradient-related MDD patients (all AUCs were more than 0.80) except for MDDmSI from MDDmoSI patients (AUC = 0.73). Detailed information is described in Fig. 4 and Supplementary Table 7.

### Effects of age and sex on the networks and clinical variables

**Fig. 5:**
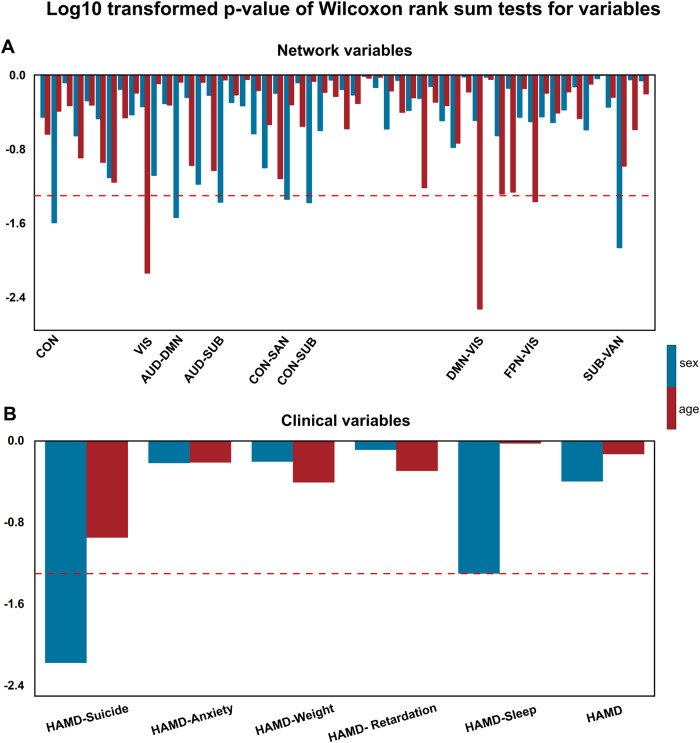
Sex and age effects on networks and clinical variables in MDD patients. Log10 transformed *p*-values of Wilcoxon rank sum tests for network variables (**A**) and clinical variables (**B**), between females and males, and between younger (age: 18–37) and older (age: 38–65) in MDD patients. The red dashed lines represent a cut-off value with a log10 transformed *p*-value = 0.05:log100.05 ≈ −1.301. Below red dashed line below showed significant effects of sex and age on the three variables. All abbreviations can be found in Supplementary Table 8 in the Supplement.

Wilcoxon rank sum tests were used to determine the potential effects of age and sex on large-scale brain networks and clinical variables in females and males, or in younger and older participants, separately. The current study established that age and sex had significantly different impacts on the two variables (Fig. 5). Specifically, females and males displayed distinctive effects on WNC and BNC in large-scale networks, especially in CON, AUD-DMN, AUD-SUB, CON-SAN, CON-SUB, and SUB-VAN, whereas younger and older subjects displayed differential effects on VIS, VIS-DMN, and VIS-FPN. In addition, clinical performance including suicide and sleep disorder was preferably targeted by females. These findings initially showed that the potential heterogeneity of large-scale networks and clinical variables is related to age and sex.

### Validation

The current study repeated these analyses to validate current findings in independent cohorts that included new 499 HCs and the original 528 MDD patients. More females and lower educational years were found in MDD subgroup patients compared with the new HC group. In addition, large-scale network roles analysis showed similar network dynamics in MDD subgroups compared with the HC validation group (Supplementary Table 3). More importantly, MDD subgroups showed similar differential WNC, BNC, and one-versus-all other network connectivity compared with the HC validation group. Detailed information is described in Supplementary Fig. 6. Further, 31 pairs of network connections used to perform classification analysis with the SVM approach also showed similar findings (Supplementary Fig. 7).

## Discussion

For the first time, the current study demonstrated that part neural basis of the suicidality gradient in MDD patients was the perturbations in the whole-brain connectome. The altered brain connections represent powerful diagnostic biomarkers that can discriminate MDD with or without suicidal ideas and behavior. These findings provided novel insights for understanding brain correlates of mild to severe suicidal symptoms in depression and significantly advanced assessment of which MDD patients are at greatest risk of suicide.

The findings of the current study corroborate previous findings and extend them in three important ways. First, the findings of the current study have implications for understanding how the dynamic trajectory of large-scale FC network roles impacts brain architectures with a high degree of connectivity, which is critical for regulating the flow and integration of information underlying suicidality in MDD patients. On one hand, although suicide-related brain structural and functional changes in the prefrontal-temporal-limbic system were frequently reported, there is an ongoing debate and inconsistent options on the association between brain network features and suicidality in MDD patients. A recent meta-analysis including 45 neuroimaging studies established that gray matter atrophy, white matter disintegration, and network disruption within the frontal-temporal system were the strongest correlates of suicide attempts in MDD patients [37], whereas reduced FC in frontal-temporal system was associated with discriminating gradual susceptibility of suicidal idea in MDD patients [38]. In addition, accumulating research evidence showed that reduced orbitofrontal-thalamic FC and disrupted frontal-subcortical WM integrity were related to suicidal ideation in MDD patients [17, 18]. Furthermore, previous studies aver that reduced dynamic amplitude of low-frequency fluctuation in the orbital frontal cortex, dorsal anterior cingulate cortex, left inferior temporal gyrus, and left hippocampus gyrus could serve as predictive biomarkers of SI severity in MDD patients [39]. Notably, MDD with SB patients showed differential activation patterns in the prefrontal cortex-limbic system when performing emotional or cognitive tasks, indicating heterogeneity of suicide in depression [11]. These disagreements in the location of findings and nature of connectivity changes [17, 18, 39], which map connectional abnormalities of structure and functional networks associated with suicidality in depression, might be due to the small sample size, thereby limiting the generalizability of findings. Therefore, it is plausible that recruiting large sample sizes to explore common and specific network basis of suicidality revealed neuroimaging-informed phenotype of suicidality in MDD patients. On the other hand, analysis of a large multi-centric dataset of individuals with MDD in the current study showed perturbed functional networks, especially in DMN, VAN, SMN, and DAN [23], and reduced temporal variations, indicating abnormal communications between large-scale brain networks over time in MDD patients [11]. The current study established disturbance of within- and between large-scale network connectivity, which has been reported consistently by previous neuroimaging studies [40–43] and recent large-scale meta-analyses [44]. However, the current study findings also showed that dynamic changes in network roles were less frequently reported at large-scale network levels, especially in FPN, CON, SUB, VAN, AUD, SUB-VAN, and AUD-SMN. These abnormal WNCs and BNCs still existed among group-level comparisons and within MDD subgroup patients. Notably, these dynamic trajectory changes in AUD, FPN, CON, and VAN have been found in previous studies to be associated with goal-oriented attention deficits, maladaptive rumination, and suicidality [45, 46]. These findings indicated that interactive links within- and between networks mutually modulate behavioral heterogeneity, depending on the nature of their functional link, whereas imbalanced within- and between networks may lead to cognitive impairment, attentional deficits, emotional dysregulation, and suicidality, which characterize MDD [47, 48]. Furthermore, these findings strongly indicate that processes of suicidal ideas or acts depend on large-scale network balance, instead of one network alone.

Second, previous studies have indicated that dynamic coalitions of large-scale networks consisting of brain areas may be engaged in complex cognitive-emotional behaviors [48, 49]. Network connectivity has consistently been found to be associated with clinical phenotype and disease severity [49–51], and emerged as a potential intermediate phenotype biomarker for mental disorders [52]. Several previous studies on brain network architectures have implicated network connectivity in depressive or anxiety symptoms determined by distinctive clinical scales [51, 53, 54]. Notably, the current study previously reported that those alterations in brain networks especially in DMN were correlated with the use of medication whereas DMN connectivity was positively correlated with symptom severity in recurrent MDD [23]. More attention has recently focused on the examination of the association of brain network features and suicidality-related behavior and established that structural atrophy and functional disruption of brain networks were significantly associated with differential suicidality in MDD patients [13, 14, 46, 55–57]. In addition, a new network-based framework recently proposed that functional network alterations, especially in FPN and DMN networks, differentially distinguished suicide attempters from suicide ideators in depressed patients [58]. In the current study, these findings were extended from two facts: differential brain correlates were simultaneously associated with specific clinical symptoms, and distinctive network connections were involved in the suicidality gradient in depression. These symptom-specific changes of within- and between-network connectivity in suicidality-related MDD patients highlight the heterogeneity of suicide in depression and indicate that loss of balance for these network links promotes the occurrence of specific behavior, including suicidal ideas or acts. More recently, structural brain measures were shown in previous studies to link with clinical phenotypes and showed hidden dimensions of brain-behavior relationships in MDD patients and were replicated across clinical centers [59]. As a result, disrupted large-scale within- and between networks coupled together and synergistically tilted network imbalance towards specific behavior, including suicide. Nevertheless, these imbalanced network links may characterize pathological states and facilitate the activation of the metaphorical switches to make subjects more vulnerable to producing and maintaining suicidal ideas or behavior under depressive conditions.

Third, the current study established that the 31 pairs of overlapping network connections were simultaneously associated with suicidality gradient in depression and provided a more reliable tool for diagnostic identification prediction. Based on the findings of the current study, mean FCIs in these regions were more sensitive in identifying one subject in normal or MDDSB patients compared with MDD patients with or without SI. On the other hand, these FCIs are superior in the prediction of MDD with SI or SB compared with MDD without SI. In addition, even within MDD with SI or SB, these FCIs are still powerful tools to discriminate MDD with SI or SB, except MDDmSI from MDDmoSI (AUC = 0.73). These findings indicate that the use of large-scale network connection approaches to identify more robust diagnostic neuroimaging biomarkers may vary depending on whether the prediction goal pertains to diagnosis. Therefore, the established key features of large-scale network dynamics are crucial for early recognition and timely diagnosis of individuals with suicidality in depression and achieve much greater progress towards understanding and preventing suicide, as well as reducing patients’ risk of morbidity from suicide ideation and attempts and their risks of suicide death. In clinical translational practice, measured imbalanced network links in the current study served as functional endophenotypes to particularly characterize depressive patients who tend to disguise real suicide intent without apparent symptoms. In addition, the current study used this endophenotype to guide informed treatment and monitor if medications target these networks.

Several previous studies have established the effects of age and gender on large-scale network dynamics in healthy and depressive disorders [60–62]. In most previous studies, age and gender, taken as covariates of no interest, were controlled to avoid potential effects on brain network analysis. The current study established that age and gender had different effects on large-scale networks and clinical variables, as shown in Fig. 5. This finding indicated that caution should be observed when controlling for effects of age and gender under some conditions, including depression.

The current study had some limitations. First, this was a cross-sectional study involving multiple centers. A longitudinal study should be undertaken to validate these findings. Second, the suicidality gradient in depression was assessed using HAMD suicidal factor scores, which may limit findings. Therefore, the use of improved suicidality assessment instruments is necessary to precisely evaluate the severity of suicide in MDD patients in future studies. Third, future studies are needed to establish whether these abnormal networks are long-lasting and how they may interact with environmental and genetic factors.

In conclusion, the current study demonstrated that the dynamic trajectory of network roles at a large-scale level was associated with suicidality gradient in MDD patients. Abnormal overlapping network connections were used as neuroimaging biomarkers in the diagnostic identification of subjects who are vulnerable to suicide under depressive conditions. The current study achieved much greater progress towards understanding the pathologic mechanism of suicide and precisely preventing suicidal occurrence via targeting these circuits with effective medical or physical instruments.

### Supplementary information

supplemental material

## Data Availability

Data of the REST-meta-MDD project are available at: 10.57760/sciencedb.o00115.00013. A combination of MATLAB-based (Mathworks Inc., Natick, MA, USA) software packages were used to perform all statistical analyses.
