# Supplementary material for: Connectomics-based resting-state functional network alterations predict suicidality in major depressive disorder
Source: Transl Psychiatry. 2023 Nov 27;13:365. doi: 10.1038/s41398-023-02655-4 (PMC10682490; doi:10.1038/s41398-023-02655-4)
Supplement: Supplementary file 1 — supplemental material [file 41398_2023_2655_MOESM1_ESM.docx]

**Supplemental Material**

**These supplemental materials include:**

**METHODS**

**RESULTS**

**STable 1** The contributing sample size, clinical information of patients, and key data acquisition parameters of each study center included in the current study.

**STable 2** Demographic and clinical characteristics of the subjects.

**STable 3** Demographic and clinical characteristics for validation group subjects.

**STable 4.** Network variables within the subgroups of MDD.

**STable 5** Thirty-one functional connection links used for classification between groups.

**STable 6** Correlation analysis revealed correlation coefficients and p values between large-scale network connectivity and clinical variables in the MDD subgroup patients.

**STable 7** Classification performance in the experimental datasets and validation datasets by SVM.

**STable 8** List of abbreviations used in figures and tables.

**SFig. 1** Network roles.

**SFig. 2** Differential network connections among the ten RSNs in the MDD patients compared to healthy controls.

**SFig. 3** Divergent network connections among the ten RSNs in the suicidality related MDD patients compared to MDD patients without suicidality.

**SFig. 4** Divergent network connections among the ten RSNs within suicidality related MDD patients.

**SFig. 5** Common connections of group-level comparison among the ten RSNs in the five sub-groups of MDD patients and healthy controls.

**SFig. 6** Network roles of validation group.

**SFig. 7** Neuroimaging biomarker for classifying validation group and MDD patients.

**METHODS**

**Study subjects and selection criteria**

A vast majority of data in the present study was obtained from the REST-meta-MDD project, which currently includes 1300 patients with major depressive disorder (MDD) and 1128 healthy controls (HC) from 17 sites across China [1]. We also recruited a separate group of 23 MDD patients with suicidal behavior, from the Department of Psychiatry at Henan Provincial Mental Hospital. All participants were right-handed Han Chinese. Details about this project can be found elsewhere [1], and the dataset is publicly available at <http://rfmri.org/REST-meta-MDD>. A total of 1323 MDDs and 1128 HCs were recruited in the study, based on the following selection criteria: 1) aged between 18-65 years; 2) MDD patients had complete 17-item Hamilton Depression Scale (HAMD) information; 3) drug naïve or drug free for longer than three weeks. Among them, 562 and 1127 MDD patients and HCs, respectively, met the above criteria, and were therefore subjected to analysis. Notably, we excluded 48 cases (24 MDD and 24 HCs) due to data duplication, as well as 10 MDD patients and 105 HCs because of excessive head motion or poor data quality (see details in Data Acquisition, Preprocessing and Quality Control).

Finally, a total of 528 MDD patients and 998 HCs were enrolled in the analysis. We calculated scores of 5 factors of 17-item HAMD, namely suicide, anxiety, weight, retardation, and sleep. The suicide factor scores were classified as follows; 0 points - major depressive disorder without suicidal ideation (MDDNSI, n = 134), 1 point- major depressive disorder with mild suicidal ideation (MDDmSI, n = 150), 2 points- major depressive disorder with moderate suicidal ideation (MDDmoSI, n = 110), 3 points- major depressive disorder with severe suicidal ideation (MDDSSI, n = 93), and 4 points- major depressive disorder with suicidal behavior (MDDSB, n = 41). Finally, we used *randperm.m* in MATLAB to randomly divide healthy controls into HCs (HC, n = 499) and verification (n = 499) groups.

**Data acquisition, preprocessing, and quality control**

Resting-state fMRI and structural T1-weighted MRI brain scans were acquired at each site and locally preprocessed using the DPARSF software, according to a standardized protocol [2]. Briefly, the procedure involved removal of the first 10 volumes for signal equilibrium, slice-timing correction, head motion realignment, brain tissue segmentation, spatial normalization, and temporal filtering (0.01-0.10Hz). To control for head motion and physiological noises, regressed out the Friston-24 head motion parameters [3], liner trend, as well as signals from the white matter, cerebrospinal fluid and whole brain. Thereafter, we performed global signal regression here, in line with recent studies which have suggested that the temporal variability of functional connectivity (FC) is sensitive to artifacts induced by head motion [4], and global signal regression is effective for mitigating this problem [5]. Additionally, we calculated the mean framewise-displacement (FD) [6], to exclude subjects with excessive head motion and address the residual effects of head motion as a covariate in group analyses.

Consequently, a total of 10 MDD patients and 105 HCs were excluded because of excessive head motion or poor data quality. Among them, 8 MDD patients and 87 HCs were excluded due to poor imaging quality or inaccurate spatial normalization, after a careful manual checking, 1 MDD patient and 10 HCs were excluded because of excessive head motion, based on a mean FD > 0.2 mm, whereas 1 MDD patient and 8 HCs were excluded for bad coverage (fMRI signal was lost in any regions of interest (ROIs) from the Power atlas). The final sample for analysis, after exclusion of these subjects, consisted of 528 and 998 MDD patients and HCs respectively.

**Construction of functional networks**

First, we applied the Power Atlas[7] to partition the brain of each participant into 264 cortical and subcortical ROIs. For each participant, whole-brain functional connectivity, between all pairs of brain regions, was constructed from the preprocessed fMRI data. Notably, 226 out of the 264 ROIs have been previously assigned to 10 well-established large-scale resting-state networks (RSNs) [7-9], including the auditory network (AUD), the cingulo-opercular task control network (CON), the dorsal and ventral attention network (DAN and VAN), the default mode network (DMN), the fronto-parietal task control network (FPN), the salience network (SN), the sensorimotor network (SMN), the subcortical network (SUB), and the visual network (VIS). In addition, we estimated FC between all pairs of the 226 ROIs for each subject using the Wavelet coherence and generated a 226×226 connectivity matrix for each participant.

**Functional connectivity within- and between RSNs**

Three types of network connectivity between two ROIs for the 10 RSNs, namely within-network, one-versus-all-others-network, and pairwise network, were computed onto the connectivity matrices as follows:


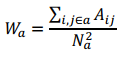


Firstly, we adopted the definition of [10], to calculate within-network connectivity (W𝑎) for each RSN (𝑎 ∈{1,2, ⋯,10} ) as the average connectivity across all the links within the RSN normalized by the square of the number of nodes (ROIs) within the network 𝑎, as follows:

Where A _i j_ is the 226×226 connectivity matrix; N𝑎 denotes the number of nodes within the network 𝑎; while 𝑖 and 𝑗 denote the power ROIs.

Secondly, for a network with N nodes (here, N = 226), we computed a one-versus-all-others-network connectivity (B𝑎−𝜀) as the average connectivity across all the links from one reference RSN (𝑎 ∈ {1,2, ⋯,10}) to all other RSNs (𝜀 ∈ {1,2, ⋯,10} − 𝑎), normalized by the product of the number of nodes within the reference network 𝑎 and all other networks, as follows:


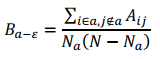


Thirdly, we computed the pairwise connectivity between networks (PB𝑎-𝑏), as the average connectivity across all the links between two RSNs, 𝑎 and 𝑏 (𝑎, 𝑏 ∈ {1,2, ⋯,10}; 𝑎 ≠ 𝑏), normalized by the product of the number of nodes within the two networks, as follows:


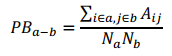


To investigate the functional roles of 10 RSNs defined by Power et al. [7], we calculated the within- and the between-network connectivity to map out a 2-dimensional space (first introduced by Bassett et al. [10]. To this end, we adopted a procedure conceptually akin to previously defined methods for network cartography [11]. Briefly, within-network connectivity is calculated as the average connectivity/strength across all the links within the RSN normalized by the square of the number of nodes (ROIs) within the network. Instead of focusing on the relative proportion of within-network connections, we directly targeted the strength of connectivity between networks. We therefore chose the between-network connectivity as the second dimension of the 2D plane. Since these choices appear relatively arbitrary, we therefore chose to define 4 quadrants of the 2D plane based on whether the within-and between-network connectivity was greater/lesser than the average.

All statistical analyses were performed using a combination of MATLAB-based (MathWorks Inc., Natick, MA, USA) software packages.

**RESULTS**

**Demographic Information in MDD Patients**

A summary of demographic and clinical information on the study cohorts of all MDD patients is outlined in **STable 1**. Results of the subgroup analyses revealed significant differences in sex and years of education, but not in age, between MDD patients and HC group All these variables were controlled as covariates of no interest in subsequent analyses.

**Group-level differences of network connectivity in MDD patients relative to healthy controls**

Results of similar analyses investigating the functional roles of 10 RSNs overall MDD group are shown in **SFig. 1C**. Summarily, distributions of network roles were similar across the five sub-groups of MDD patients. In addition, we assessed the dynamic trajectory of averaged FC values within- and between networks, between MDD patients and HCs (**SFig. 1D**), then, we presented the matrices of ten RSNs (**SFig. 1F**) and evaluated the differences according to the averaged within-network connectivity (WNC) as well as pairwise between-network connectivity (BNC), between MDD patients and HC group (**SFig. 1G**). Summarily, MDD patients had significantly different WNC in the AUD, CON and SUB, and BNC in the AUD-SMN, DAN-VAN and SUB-VAN, compared to HCs. In addition, we observed significant differences in all network connectivity links between the groups (**SFig. 1H**).

**Mapping the abnormal network connections among the ten RSNs in all MDD patients**

All MDD patients exhibited significantly higher and lower network connections, relative to HCs (**SFig. 2**).

**References**

1. Yan CG, Chen X, Li L, Castellanos FX, Bai TJ, Bo QJ *et al.* Reduced default mode network functional connectivity in patients with recurrent major depressive disorder. *Proc Natl Acad Sci U S A* 2019;116:9078-9083.

2. Yan C, Zang Y. DPARSF: a MATLAB toolbox for "pipeline" data analysis of resting-state fMRI. *Frontiers in Systems Neuroscience* 2010;4:13.

3. Friston KJ, Williams S, Howard R, Frackowiak RSJ, Turner R. Movement-Related effects in fMRI time-series. *Magnetic Resonance in Medicine* 1996;35:346-355.

4. Laumann TO, Snyder AZ, Mitra A, Gordon EM, Gratton C, Adeyemo B *et al.* On the Stability of BOLD fMRI Correlations. *Cerebral Cortex* 2017;27:4719-4732.

5. Lydon-Staley DM, Ciric R, Satterthwaite TD, Bassett DS. Evaluation of confound regression strategies for the mitigation of micromovement artifact in studies of dynamic resting-state functional connectivity and multilayer network modularity. *Network Neuroscience* 2018;3:427-454.

6. Jenkinson M, Bannister P, Brady M, Smith S. Improved Optimization for the Robust and Accurate Linear Registration and Motion Correction of Brain Images. *NeuroImage* 2002;17:825-841.

7. Power JD, Cohen AL, Nelson SM, Wig GS, Barnes KA, Church JA *et al.* Functional network organization of the human brain. *Neuron* 2011;72:665-678.

8. Cole MW, Reynolds JR, Power JD, Repovs G, Anticevic A, Braver TS. Multi-task connectivity reveals flexible hubs for adaptive task control. *Nat Neurosci* 2013;16:1348-1355.

9. Mohr H, Wolfensteller U, Betzel RF, Misic B, Sporns O, Richiardi J *et al.* Integration and segregation of large-scale brain networks during short-term task automatization. *Nat Commun* 2016;7:13217.

10. Gu S, Satterthwaite TD, Medaglia JD, Yang M, Gur RE, Gur RC *et al.* Emergence of system roles in normative neurodevelopment. *Proc Natl Acad Sci U S A* 2015;112:13681-13686.

11. Guimerà R, Nunes Amaral LA. Functional cartography of complex metabolic networks.

**STable 1.** The contributing sample size, clinical information of patients, and key data acquisition parameters of each study center included in the current study.

| Site | Institutions | MDD | HC | Scanner | Receive  (coil) | TR (ms) | TE (ms) | Time points |
| --- | --- | --- | --- | --- | --- | --- | --- | --- |
| 1 | National Clinical Research Center for Mental Disorders (Peking University Sixth Hospital) & Key Laboratory of Mental Health, Ministry of Health (Peking University) | 72 | 72 | Siemens Tim Trio 3T | 32 channels | 2000 | 30 | 210 |
| 2 | Department of Clinical Psychology, Suzhou Suzhou Psychiatric Hospital, The Affiliated Guangji Hospital of Soochow University | 29 | 30 | Philips Achieva 3T | 8 channels | 2000 | 30 | 200 |
| 3 | The Second Xiangya Hospital of Central South University | 0 | 31 | Siemens Magnetom Symphony scanner 1.5 T | 16 channels | 2000 | 40 | 150 |
| 4 | Department of Psychiatry, Shanghai Jiao Tong University School of Medicine | 0 | 11 | GE Signa 3T | 32 channels | 3000 | 30 | 100 |
| 5 | Department of Psychiatry, Shanghai Jiao Tong University School of Medicine | 15 | 15 | Siemens Tim Trio 3T | 32 channels | 2000 | 30 | 180 |
| 6 | Sir Run Run Shaw Hospital, Zhejiang University School of Medicine | 36 | 48 | GE discovery MR750 | 8 channels | 2000 | 30 | 184 |
| 7 | Department of Psychiatry, First Affiliated Hospital, China Medical University | 57 | 61 | GE Signa 3T | 8 channels | 2000 | 30 | 200 |
| 8 | The First Affiliated Hospital of Jinan University | 0 | 50 | GE Discovery MR750 3.0T | 8 channels | 2000 | 25 | 200 |
| 9 | First Hospital of Shanxi Medical University | 0 | 26 | Siemens Tim Trio 3T | 32 channels | 2000 | 30 | 212 |
| 10 | Department of Psychiatry, The First Affiliated Hospital of Chongqing Medical University | 7 | 29 | GE Signa 3T | 8 channels | 2000 | 30 | 200 |
| 11 | Department of Psychiatry, The First Affiliated Hospital of Chongqing Medical University | 32 | 6 | GE Signa 3T | 8 channels | 2000 | 30 | 240 |
| 12 | The First Affiliated Hospital of Xi’an Jiaotong University, Xi’an Central Hospital | 0 | 15 | GE Excite 1.5T | 16 channels | 2500 | 35 | 150 |
| 13 | The Second Xiangya Hospital of Central South University | 61 | 31 | Siemens Tim Trio 3T | 32 channels | 2500 | 25 | 200 |
| 14 | Department of Psychosomatics and Psychiatry, Zhongda Hospital, School of Medicine, Southeast University | 0 | 49 | Siemens Verio 3.0T MRI | 12 channels | 2000 | 25 | 240 |
| 15 | Huaxi MR Research Center, West China Hospital of Sichuan University | 0 | 31 | GE Signa 3T | 8 channels | 2000 | 30 | 200 |
| 16 | Department of Psychiatry, The First Affiliated Hospital of Chongqing Medical University | 0 | 41 | GE Signa 3T | 8 channels | 2000 | 40 | 240 |
| 17 | Department of Radiology, The First Affiliated Hospital, College of Medicine, Zhejiang University | 21 | 20 | Philips Achieva 3.0 T scanner | 8 channels | 2000 | 35 | 200 |
| 18 | Anhui Medical University | 50 | 36 | GE Signa 3T | 8 channels | 2000 | 22.5 | 240 |
| 19 | Faculty of Psychology, Southwest University | 0 | 251 | Siemens Tim Trio 3T | 12 channels | 2000 | 30 | 242 |
| 20 | Beijing Anding Hospital, Capital Medical University | 79 | 59 | Siemens Tim Trio 3T | 32 channels | 2000 | 30 | 240 |
| 21 | The Institute of Mental Health, Second Xiangya Hospital of Central South University | 23 | 19 | Philips Gyroscan Achieva 3.0T | 32 channels | 2000 | 30 | 250 |
| 22 | Mental Health Center, West China Hospital, Sichuan University | 23 | 28 | Philips Achieva 3.0T TX | 8 channels | 2000 | 30 | 240 |
| 23 | Department of Neurology, Affiliated ZhongDa Hospital of Southeast University | 0 | 39 | Siemens Verio 3T | 12 channels | 2000 | 25 | 240 |
| 24 | Department of Psychiatry at Henan Provincial Mental Hospital | 23 | 0 | Siemens Verio 3T | 12 channels | 2000 | 25 | 240 |

Abbreviations: MDD, major depressive disorder; HC, healthy control.

**STable 2.** Demographic and Clinical Characteristics for All Subjects.

| Measures | HC  (n=499) | MDD  (n=528) | p-values |
| --- | --- | --- | --- |
| Age (years) | 33.1 ± 12.6 | 33.2 ± 11.2 | p = 0.85^a^ |
| Sex (%female) | 297 (55.80%) | 344 (63.35%) | p = 0.005^b^ |
| Education (years) | 13.9 ± 3.5 | 12.0 ± 3.5 | p < 0.0001^a^ |
| HAMD | n.a. | 21.5 ± 7.3 | n.a. |

Notes: Unless otherwise indicated, data are presented as mean ± standard deviation. ^a^ Differences in these variables between MDD patients and normal controls were tested using two-sample two-tailed t tests (p < .05); ^b^ Differences in the sex distribution between groups were tested using the chi-squared (χ2) test (p < .05). All abbreviations can be found in **STable 8**.

**STable 3.** Demographic and Clinical Characteristics for Validation Group Subjects.

| **Groups** | **HC**  **(Validation,**  **n=499)** | **MDD** | | | | | **F/X^2^**  **values** | **P**  **values** |
| --- | --- | --- | --- | --- | --- | --- | --- | --- |
|  |  | **MDDNSI**  **(n=134)** | **MDDmSI**  **(n=150)** | **MDDmoSI**  **(n=110)** | **MDDSSI**  **(n=93)** | **MDDSB**  **(n=41)** |  |  |
| **Age (years)** | 39.71 ± 17.53 | 33.78 ± 11.68^a^ | 34.6 ± 11.48^b^ | 32.88 ± 11.06^c^ | 31.41 ± 9.94^d^ | 28.33 ± 11.70^e^ | 11.32 | p<0.001 |
| **Sex (%, female)** | 323 (60.60%) | 77 (55.40%) | 96 (63.15%) | 81 (71.68%) | 67 (67.68%) | 23 (53.49%) | 10.36 | 0.066^†^ |
| **Education (years)** | 17.53 ± 3.43 | 11.79 ± 3.40^a^ | 11.87 ± 3.72^b^ | 12.11 ± 3.52^c^ | 12.38 ± 3.33 | 11.23 ± 3.39^e^ | 6.30 | p<0.001 |

Notes: ^†^, *p* value was obtained by chi-square test; other *p* values were obtained by analyses of variance (ANOVA) among groups. Unless otherwise indicated, data are presented as the mean ± standard deviation. Post-hoc analyses were used with least significance difference (LSD) for multiple group comparison correction (*P*<0.05): ^a^, statistical difference was detected between validation group and MDDNSI group; ^b^, statistical difference was detected between validation group and MDDmSI group; ^c^, statistical difference was detected between validation group and MDDmoSI group; ^d^, statistical difference was detected between validation group and MDDSSI group; ^e^, statistical difference was detected between validation group and MDDSB group. All abbreviations can be found in **STable 8.**

**STable 4. Network variables within the subgroups of MDD.**

| **Network variables** | **MDDNSI** | **MDDmSI** | **MDDmoSI** | **MDDSSI** | **MDDSB** | **P value** |
| --- | --- | --- | --- | --- | --- | --- |
| **Within-network connectivity** | | | | | |  |
| **AUD** | 0.136 ± 0.067 | 0.147 ± 0.07 | 0.153 ± 0.071 | 0.143 ± 0.067 | 0.13 ± 0.064 | 0.31 |
| **CON** | 0.157 ± 0.081 | 0.182 ± 0.083 | 0.191 ± 0.08 | 0.178 ± 0.078 | 0.174 ± 0.068 | 0.014 |
| **DAN** | 0.112 ± 0.062 | 0.123 ± 0.069 | 0.122 ± 0.064 | 0.119 ± 0.057 | 0.114 ± 0.053 | 0.58 |
| **DMN** | 0.102 ± 0.042 | 0.109 ± 0.043 | 0.11 ± 0.043 | 0.105 ± 0.041 | 0.109 ± 0.04 | 0.569 |
| **FPN** | 0.121 ± 0.046 | 0.125 ± 0.055 | 0.13 ± 0.048 | 0.128 ± 0.047 | 0.109 ± 0.045 | 0.373 |
| **SAN** | 0.128 ± 0.057 | 0.153 ± 0.072 | 0.147 ± 0.056 | 0.142 ± 0.056 | 0.141 ± 0.076 | 0.018 |
| **SMN** | 0.125 ± 0.07 | 0.13 ± 0.079 | 0.141 ± 0.066 | 0.138 ± 0.071 | 0.132 ± 0.094 | 0.423 |
| **SUB** | 0.147 ± 0.083 | 0.166 ± 0.082 | 0.177 ± 0.072 | 0.177 ± 0.079 | 0.143 ± 0.077 | 0.01 |
| **VAN** | 0.107 ± 0.065 | 0.118 ± 0.068 | 0.12 ± 0.057 | 0.114 ± 0.054 | 0.091 ± 0.061 | 0.214 |
| **VIS** | 0.203 ± 0.103 | 0.211 ± 0.102 | 0.213 ± 0.088 | 0.21 ± 0.096 | 0.22 ± 0.094 | 0.907 |
| **One-versus-all-others network connectivity** | | | | | |  |
| **AUD-versus-all-others** | 0.02 ± 0.024 | 0.022 ± 0.024 | 0.023 ± 0.026 | 0.023 ± 0.028 | 0.016 ± 0.022 | 0.647 |
| **CON-versus-all-others** | 0.016 ± 0.021 | 0.02 ± 0.027 | 0.022 ± 0.027 | 0.019 ± 0.024 | 0.015 ± 0.024 | 0.32 |
| **DAN-versus-all-others** | 0.003 ± 0.027 | 0.008 ± 0.027 | 0.008 ± 0.025 | 0.008 ± 0.025 | 0.005 ± 0.021 | 0.413 |
| **DMN-versus-all-others** | 0.081 ± 0.041 | 0.088 ± 0.045 | 0.09 ± 0.042 | 0.083 ± 0.042 | 0.084 ± 0.041 | 0.401 |
| **FPN-versus-all-others** | 0.041 ± 0.033 | 0.043 ± 0.035 | 0.046 ± 0.032 | 0.042 ± 0.035 | 0.051 ± 0.031 | 0.623 |
| **SAN-versus-all-others** | 0.002 ± 0.023 | 0.004 ± 0.028 | 0.001 ± 0.026 | 0.004 ± 0.025 | 0.006 ± 0.028 | 0.834 |
| **SMN-versus-all-others** | 0.005 ± 0.022 | 0.005 ± 0.019 | 0.01 ± 0.023 | 0.006 ± 0.021 | 0.013 ± 0.026 | 0.202 |
| **SUB-versus-all-others** | 0.002 ± 0.034 | 0 ± 0.031 | 0.002 ± 0.032 | 0.002 ± 0.036 | 0.011 ± 0.03 | 0.501 |
| **VAN-versus-all-others** | 0.077 ± 0.069 | 0.087 ± 0.069 | 0.099 ± 0.061 | 0.1 ± 0.063 | 0.078 ± 0.06 | 0.038 |
| **VIS-versus-all-others** | 0.037 ± 0.038 | 0.04 ± 0.042 | 0.04 ± 0.035 | 0.039 ± 0.039 | 0.036 ± 0.031 | 0.968 |
| **Pairwise network connectivity** | | | | | |  |
| **AUD-CON** | 0.225 ± 0.118 | 0.258 ± 0.136 | 0.272 ± 0.145 | 0.251 ± 0.122 | 0.235 ± 0.124 | 0.064 |
| **AUD-DAN** | 0.068 ± 0.094 | 0.078 ± 0.1 | 0.07 ± 0.11 | 0.072 ± 0.101 | 0.077 ± 0.08 | 0.93 |
| **AUD-DMN** | 0.114 ± 0.076 | 0.129 ± 0.09 | 0.127 ± 0.094 | 0.122 ± 0.078 | 0.118 ± 0.079 | 0.588 |
| **AUD-FPN** | 0.048 ± 0.073 | 0.046 ± 0.08 | 0.061 ± 0.09 | 0.051 ± 0.084 | 0.048 ± 0.083 | 0.65 |
| **AUD-SAN** | 0.078 ± 0.083 | 0.093 ± 0.099 | 0.077 ± 0.096 | 0.076 ± 0.083 | 0.064 ± 0.086 | 0.418 |
| **AUD-SMN** | 0.151 ± 0.1 | 0.167 ± 0.113 | 0.18 ± 0.116 | 0.178 ± 0.114 | 0.156 ± 0.1 | 0.208 |
| **AUD-SUB** | 0.072 ± 0.1 | 0.091 ± 0.107 | 0.084 ± 0.114 | 0.087 ± 0.098 | 0.06 ± 0.098 | 0.493 |
| **AUD-VAN** | 0.066 ± 0.151 | 0.085 ± 0.165 | 0.087 ± 0.156 | 0.101 ± 0.128 | 0.071 ± 0.129 | 0.499 |
| **AUD-VIS** | 0.033 ± 0.102 | 0.03 ± 0.101 | 0.039 ± 0.111 | 0.029 ± 0.093 | 0.018 ± 0.085 | 0.908 |
| **CON-DAN** | 0.098 ± 0.107 | 0.095 ± 0.108 | 0.107 ± 0.116 | 0.095 ± 0.1 | 0.124 ± 0.094 | 0.723 |
| **CON-DMN** | 0.163 ± 0.101 | 0.18 ± 0.104 | 0.186 ± 0.104 | 0.175 ± 0.096 | 0.177 ± 0.089 | 0.464 |
| **CON-FPN** | 0.01 ± 0.074 | 0.004 ± 0.077 | 0.017 ± 0.09 | 0.008 ± 0.088 | 0.01 ± 0.076 | 0.807 |
| **CON-SAN** | 0.198 ± 0.113 | 0.235 ± 0.118 | 0.229 ± 0.107 | 0.22 ± 0.109 | 0.217 ± 0.139 | 0.066 |
| **CON-SMN** | 0.139 ± 0.083 | 0.153 ± 0.107 | 0.165 ± 0.1 | 0.156 ± 0.102 | 0.135 ± 0.102 | 0.264 |
| **CON-SUB** | 0.132 ± 0.102 | 0.159 ± 0.113 | 0.152 ± 0.113 | 0.154 ± 0.102 | 0.135 ± 0.105 | 0.251 |
| **CON-VAN** | 0.066 ± 0.148 | 0.079 ± 0.169 | 0.095 ± 0.166 | 0.096 ± 0.141 | 0.049 ± 0.143 | 0.408 |
| **CON-VIS** | 0.019 ± 0.095 | 0.022 ± 0.109 | 0.001 ± 0.107 | 0.022 ± 0.096 | 0.024 ± 0.078 | 0.383 |
| **DAN-DMN** | 0.138 ± 0.084 | 0.148 ± 0.084 | 0.145 ± 0.082 | 0.132 ± 0.085 | 0.153 ± 0.096 | 0.549 |
| **DAN-FPN** | 0.049 ± 0.084 | 0.04 ± 0.07 | 0.043 ± 0.078 | 0.048 ± 0.073 | 0.028 ± 0.075 | 0.707 |
| **DAN-SAN** | 0.016 ± 0.091 | 0.002 ± 0.103 | 0.01 ± 0.092 | 0.006 ± 0.087 | 0.033 ± 0.109 | 0.2 |
| **DAN-SMN** | 0.084 ± 0.095 | 0.094 ± 0.094 | 0.085 ± 0.096 | 0.092 ± 0.093 | 0.091 ± 0.098 | 0.914 |
| **DAN-SUB** | 0.029 ± 0.095 | 0.036 ± 0.1 | 0.038 ± 0.099 | 0.037 ± 0.09 | 0.035 ± 0.108 | 0.953 |
| **DAN-VAN** | 0.217 ± 0.172 | 0.242 ± 0.197 | 0.271 ± 0.159 | 0.269 ± 0.161 | 0.209 ± 0.207 | 0.073 |
| **DAN-VIS** | 0.091 ± 0.114 | 0.09 ± 0.118 | 0.086 ± 0.112 | 0.068 ± 0.117 | 0.086 ± 0.117 | 0.576 |
| **DMN-FPN** | 0.036 ± 0.069 | 0.049 ± 0.072 | 0.036 ± 0.074 | 0.03 ± 0.073 | 0.046 ± 0.044 | 0.276 |
| **DMN-SAN** | 0.08 ± 0.085 | 0.095 ± 0.092 | 0.087 ± 0.091 | 0.082 ± 0.079 | 0.089 ± 0.097 | 0.632 |
| **DMN-SMN** | 0.094 ± 0.06 | 0.093 ± 0.064 | 0.107 ± 0.068 | 0.099 ± 0.066 | 0.097 ± 0.081 | 0.481 |
| **DMN-SUB** | 0.023 ± 0.068 | 0.022 ± 0.064 | 0.021 ± 0.071 | 0.021 ± 0.064 | 0.014 ± 0.079 | 0.987 |
| **DMN-VAN** | 0.027 ± 0.089 | 0.028 ± 0.102 | 0.032 ± 0.088 | 0.04 ± 0.093 | 0.038 ± 0.097 | 0.837 |
| **DMN-VIS** | 0.07 ± 0.078 | 0.075 ± 0.086 | 0.082 ± 0.082 | 0.072 ± 0.083 | 0.057 ± 0.084 | 0.662 |
| **FPN-SAN** | 0.079 ± 0.072 | 0.1 ± 0.084 | 0.099 ± 0.086 | 0.095 ± 0.068 | 0.079 ± 0.061 | 0.145 |
| **FPN-SMN** | 0.07 ± 0.079 | 0.076 ± 0.077 | 0.093 ± 0.077 | 0.089 ± 0.087 | 0.105 ± 0.099 | 0.066 |
| **FPN-SUB** | 0.019 ± 0.077 | 0.014 ± 0.074 | 0.016 ± 0.083 | 0.016 ± 0.075 | 0.036 ± 0.054 | 0.795 |
| **FPN-VAN** | 0.125 ± 0.102 | 0.129 ± 0.109 | 0.142 ± 0.103 | 0.135 ± 0.093 | 0.102 ± 0.09 | 0.474 |
| **FPN-VIS** | 0.134 ± 0.085 | 0.128 ± 0.096 | 0.136 ± 0.097 | 0.136 ± 0.083 | 0.143 ± 0.064 | 0.902 |
| **SAN-SMN** | 0.007 ± 0.087 | 0.022 ± 0.104 | 0.022 ± 0.093 | 0.025 ± 0.097 | 0.038 ± 0.127 | 0.458 |
| **SAN-SUB** | 0.088 ± 0.091 | 0.113 ± 0.105 | 0.114 ± 0.093 | 0.108 ± 0.089 | 0.106 ± 0.093 | 0.172 |
| **SAN-VAN** | 0.058 ± 0.115 | 0.061 ± 0.126 | 0.093 ± 0.119 | 0.084 ± 0.114 | 0.034 ± 0.102 | 0.048 |
| **SAN-VIS** | 0.07 ± 0.103 | 0.08 ± 0.112 | 0.064 ± 0.104 | 0.077 ± 0.107 | 0.066 ± 0.111 | 0.78 |
| **SMN-SUB** | 0.008 ± 0.114 | 0.007 ± 0.113 | 0.01 ± 0.11 | 0.01 ± 0.107 | 0.031 ± 0.122 | 0.642 |
| **SMN-VAN** | 0.065 ± 0.09 | 0.077 ± 0.103 | 0.093 ± 0.099 | 0.081 ± 0.094 | 0.073 ± 0.104 | 0.239 |
| **SMN-VIS** | 0.06 ± 0.128 | 0.063 ± 0.123 | 0.066 ± 0.113 | 0.071 ± 0.12 | 0.083 ± 0.13 | 0.924 |
| **SUB-VAN** | 0.147 ± 0.138 | 0.165 ± 0.145 | 0.193 ± 0.13 | 0.187 ± 0.149 | 0.157 ± 0.138 | 0.073 |
| **SUB-VIS** | 0.092 ± 0.113 | 0.098 ± 0.128 | 0.107 ± 0.122 | 0.076 ± 0.123 | 0.139 ± 0.121 | 0.182 |
| **VAN-VIS** | 0.089 ± 0.108 | 0.107 ± 0.124 | 0.105 ± 0.104 | 0.118 ± 0.116 | 0.103 ± 0.116 | 0.4 |

Notes: Data are presented as the mean ± standard deviation. Statistical comparisons between groups were obtained by analyses of variance (ANOVA). Post-hoc analyses were used with False Discovery Rate (FDR) for multiple group comparison correction (P < 0.05). All abbreviations can be found in **STable 8**.

**STable 5.** Thirty-One Functional Connection Links Used for Classification between Groups.

| **Thirty-one functional connections used for classification between groups** | | |
| --- | --- | --- |
| ROI 18 - ROI 216 | ROI 51 - ROI 263 | ROI 161 - ROI 233 |
| ROI 36 - ROI 39 | ROI 52 - ROI 79 | ROI 201 - ROI 203 |
| ROI 37 - ROI 108 | ROI 53 - ROI 259 | ROI 201 - ROI 204 |
| ROI 39 - ROI 209 | ROI 54 - ROI 129 | ROI 202 - ROI 208 |
| ROI 43 - ROI 229 | ROI 58 - ROI 223 | ROI 203 - ROI 205 |
| ROI 43 - ROI 238 | ROI 83 - ROI 115 | ROI 209 - ROI 208 |
| ROI 43 - ROI 256 | ROI 87 - ROI 227 | ROI 209 - ROI 210 |
| ROI 43 - ROI 259 | ROI 94 - ROI 263 | ROI 218 - ROI 263 |
| ROI 43 - ROI 263 | ROI 117 - ROI 229 | ROI 223 - ROI 233 |
| ROI 43 - ROI 264 | ROI 117 - ROI 233 |  |
| ROI 51 - ROI 215 | ROI 117 - ROI 252 |  |

Notes: Nodes of the links were from Power-atlas. All abbreviations can be found in **STable 8.**

**STable 6.** Correlation Analysis Revealed Correlation Coefficients and P Values between Large-Scale Network Connectivity and Clinical Variables in the MDD Subgroup Patients.

| Variables | HAMD-Anxiety | | HAMD-Weight | | HAMD-Retardation | | HAMD-Sleep | | HAMD | | |
| --- | --- | --- | --- | --- | --- | --- | --- | --- | --- | --- | --- |
|  | r | p | r | p | r | p | r | p | r | p | |
| MDDNSI | | | | | | | | | | | |
| FPN |  |  |  |  |  |  |  |  | -0.167 | 0.049 | |
|  | **r** | p | r | p | r | p | r | p | r | p | |
| VAN |  |  |  |  | 0.221 | 0.009 |  |  | 0.183 | 0.031 | |
| AUD-VAN |  |  | 0.209 | 0.014 |  |  |  |  |  |  | |
| AUD-VIS | 0.236 | 0.005 |  |  |  |  |  |  | 0.208 | 0.014 | |
| CON-VAN | 0.191 | 0.025 |  |  |  |  |  |  |  |  | |
| CON-VIS | 0.199 | 0.019 |  |  |  |  |  |  |  |  | |
| DAN-VIS | 0.185 | 0.029 | 0.190 | 0.025 |  |  |  |  | 0.191 | 0.025 | |
| DMN-FPN |  |  |  |  | 0.181 | 0.033 |  |  | 0.183 | 0.031 | |
| DMN-SUB | 0.171 | 0.044 |  |  | 0.188 | 0.026 | 0.195 | 0.022 | 0.215 | 0.011 | |
| DMN-VIS | -0.201 | 0.018 |  |  |  |  |  |  |  |  | |
| FPN-SAN | -0.171 | 0.044 | -0.174 | 0.041 | -0.230 | 0.006 |  |  | -0.223 | 0.008 | |
| SAN-VIS | 0.204 | 0.016 |  |  |  |  |  |  |  |  | |
| MDDmSI | | | | | | | | | | |  |
| CON | -0.177 | 0.029 |  |  |  |  |  |  |  |  | |
| SMN | -0.175 | 0.031 |  |  |  |  |  |  |  |  | |
| VIS | -0.216 | 0.007 |  |  |  |  |  |  |  |  | |
| AUD-FPN | 0.189 | 0.020 |  |  |  |  |  |  |  |  | |
| AUD-SAN | 0.165 | 0.042 |  |  |  |  |  |  |  |  | |
| AUD-SMN | -0.222 | 0.006 |  |  |  |  |  |  | -0.190 | 0.019 | |
| AUD-VAN | 0.208 | 0.010 |  |  |  |  | 0.176 | 0.031 |  |  | |
| CON-SMN | -0.219 | 0.007 |  |  |  |  |  |  | -0.208 | 0.010 | |
| DAN-SAN | 0.169 | 0.038 |  |  |  |  |  |  |  |  | |
| DAN-SMN |  |  |  |  |  |  | -0.163 | 0.045 |  |  | |
| DAN-VAN |  |  |  |  |  |  | 0.202 | 0.013 |  |  | |
| DMN-SMN |  |  |  |  |  |  | 0.159 | 0.050 | 0.175 | 0.031 | |
| DMN-VAN |  |  |  |  |  |  | -0.180 | 0.026 | -0.232 | 0.004 | |
| SAN-SUB |  |  |  |  |  |  |  |  | -0.163 | 0.044 | |
| SAN-VAN | 0.161 | 0.048 |  |  |  |  |  |  |  |  | |
| MDDmoSI | | | | | | | | | | |  |
| AUD |  |  | -0.192 | 0.041 |  |  |  |  |  |  | |
| DAN |  |  |  |  |  |  | -0.187 | 0.047 |  |  | |
| FPN |  |  | -0.216 | 0.021 |  |  |  |  |  |  | |
| SAN | -0.206 | 0.028 | -0.289 | 0.002 |  |  |  |  |  |  | |
| SMN |  |  | -0.283 | 0.002 |  |  |  |  |  |  | |
| SUB |  |  | -0.277 | 0.003 |  |  |  |  |  |  | |
| VIS |  |  | -0.216 | 0.021 |  |  |  |  |  |  | |
| AUD-SMN |  |  | -0.251 | 0.007 |  |  |  |  |  |  | |
| AUD-VAN |  |  | 0.280 | 0.003 |  |  |  |  |  |  | |
| CON-SAN |  |  | -0.215 | 0.022 |  |  |  |  |  |  | |
| CON-VAN |  |  | 0.283 | 0.002 |  |  |  |  |  |  | |
| CON-VIS | 0.237 | 0.012 |  |  |  |  |  |  | 0.190 | 0.044 | |
| DAN-VAN |  |  | 0.190 | 0.044 |  |  |  |  |  |  | |
| FPN-SAN | -0.270 | 0.004 | -0.199 | 0.035 |  |  |  |  | -0.189 | 0.044 | |
| SAN-SMN |  |  | 0.186 | 0.049 |  |  |  |  |  |  | |
| SAN-SUB |  |  | -0.222 | 0.018 |  |  |  |  |  |  | |
| SAN-VAN |  |  | 0.194 | 0.040 |  |  |  |  |  |  | |
| SAN-VIS |  |  | 0.281 | 0.003 |  |  |  |  |  |  | |
| SMN-SUB |  |  | 0.194 | 0.040 |  |  |  |  |  |  | |
| SMN-VAN | 0.234 | 0.012 | 0.213 | 0.024 |  |  |  |  |  |  | |
| SUB-VAN |  |  | 0.231 | 0.014 |  |  |  |  |  |  | |
| SUB-VIS |  |  | 0.240 | 0.010 |  |  |  |  |  |  | |
| VAN-VIS |  |  | 0.196 | 0.037 |  |  |  |  |  |  | |
| MDDSSI | | | | | | | | | | |  |
| VIS |  |  |  |  | -0.218 | 0.030 |  |  |  |  | |
| AUD-DAN |  |  | -0.203 | 0.044 |  |  |  |  |  |  | |
| AUD-FPN |  |  | -0.222 | 0.027 |  |  | -0.215 | 0.032 |  |  | |
| AUD-VAN |  |  | 0.213 | 0.034 |  |  |  |  |  |  | |
| CON-FPN |  |  | -0.202 | 0.045 |  |  | -0.265 | 0.008 |  |  | |
| DAN-DMN |  |  | 0.265 | 0.008 | 0.230 | 0.022 |  |  |  |  | |
| DAN-SMN |  |  | -0.213 | 0.034 | -0.213 | 0.034 |  |  | -0.201 | 0.046 | |
| DAN-SUB |  |  |  |  | 0.259 | 0.010 |  |  |  |  | |
| DAN-VIS |  |  |  |  | -0.226 | 0.024 |  |  |  |  | |
| DMN-SMN |  |  |  |  | 0.199 | 0.048 |  |  |  |  | |
| DMN-VIS |  |  |  |  | 0.199 | 0.048 |  |  |  |  | |
| FPN-SMN |  |  |  |  |  |  | -0.244 | 0.015 |  |  | |
| FPN-VAN | -0.227 | 0.024 |  |  |  |  |  |  |  |  | |
| SAN-SUB |  |  |  |  | -0.217 | 0.031 |  |  |  |  | |
| SAN-VAN |  |  | 0.248 | 0.013 |  |  |  |  |  |  | |
| SMN-SUB |  |  |  |  | 0.255 | 0.011 |  |  |  |  | |
| SMN-VAN |  |  |  |  |  |  |  |  | 0.231 | 0.021 | |
| SUB-VIS |  |  |  |  | 0.237 | 0.018 |  |  |  |  | |
| MDDSB | | | | | | | | | | |  |
| AUD-DAN |  |  |  |  |  |  | 0.470 | 0.037 |  |  | |
| AUD-FPN |  |  |  |  | 0.476 | 0.034 |  |  |  |  | |
| AUD-SAN |  |  |  |  |  |  |  |  | 0.459 | 0.042 | |
| CON-DAN |  |  |  |  |  |  | 0.469 | 0.037 | 0.461 | 0.041 | |
| CON-SMN |  |  |  |  |  |  | 0.573 | 0.008 |  |  | |
| DAN-FPN | 0.453 | 0.045 |  |  |  |  |  |  |  |  | |
| DMN-FPN |  |  |  |  | -0.507 | 0.023 |  |  |  |  | |
| DMN-VAN |  |  |  |  | 0.454 | 0.044 |  |  |  |  | |
| FPN-SMN |  |  |  |  |  |  |  |  | 0.491 | 0.028 | |
| SAN-SMN |  |  |  |  |  |  |  |  | 0.515 | 0.020 | |
| SMN-VIS |  |  |  |  |  |  |  |  | -0.529 | 0.016 | |
| VAN-VIS |  |  | 0.503 | 0.024 |  |  |  |  |  |  | |

All abbreviations can be found in **STable 8**.

**STable 7.** Classification performance in the experimental datasets and validation datasets by SVM.

|  | Accuracy | P value of accuracy | AUC | P value of AUC | Sensitivity | Specificity |
| --- | --- | --- | --- | --- | --- | --- |
| **Experimental datasets** |  |  |  |  |  |  |
| HC VS MDDNSI | 0.79 | < 0.001 | 0.72 | < 0.001 | 0.49 | 0.84 |
| HC VS MDDmSI | 0.78 | < 0.001 | 0.63 | < 0.001 | 0.55 | 0.66 |
| HC VS MDDmoSI | 0.82 | < 0.001 | 0.61 | < 0.001 | 0.56 | 0.62 |
| HC VS MDDSSI | 0.84 | < 0.001 | 0.65 | < 0.001 | 0.7 | 0.53 |
| HC VS MDDSB | 0.97 | < 0.001 | 0.96 | < 0.001 | 0.85 | 0.94 |
| MDDNSI VS MDDmSI | 0.49 | 0.41059 | 0.88 | < 0.001 | 0.73 | 0.89 |
| MDDNSI VS MDDmoSI | 0.48 | 0.28172 | 0.9 | < 0.001 | 0.84 | 0.86 |
| MDDNSI VS MDDSSI | 0.52 | 0.21878 | 0.91 | < 0.001 | 0.86 | 0.87 |
| MDDNSI VS MDDSB | 0.86 | 0.998 | 0.97 | < 0.001 | 0.92 | 0.88 |
| MDDmSI VS MDDmoSI | 0.54 | 0.044955 | 0.73 | < 0.001 | 0.66 | 0.74 |
| MDDmSI VS MDDSSI | 0.57 | 0.19081 | 0.8 | < 0.001 | 0.82 | 0.73 |
| MDDmSI VS MDDSB | 0.89 | < 0.001 | 0.99 | < 0.001 | 0.99 | 0.94 |
| MDDmoSI VS MDDSSI | 0.51 | 0.028971 | 0.85 | < 0.001 | 0.87 | 0.77 |
| MDDmoSI VS MDDSB | 0.82 | < 0.001 | 0.99 | < 0.001 | 0.97 | 0.94 |
| MDDSSI VS MDDSB | 0.74 | < 0.001 | 0.96 | < 0.001 | 0.99 | 0.88 |
| **Validation datasets** |  |  |  |  |  |  |
| Validation VS MDDNSI | 0.79 | < 0.001 | 0.8 | < 0.001 | 0.45 | 0.91 |
| Validation VS MDDmSI | 0.78 | < 0.001 | 0.58 | 0.001 | 0.37 | 0.78 |
| Validation VS MDDmoSI | 0.82 | < 0.001 | 0.63 | < 0.001 | 0.68 | 0.52 |
| Validation VS MDDSSI | 0.84 | < 0.001 | 0.67 | < 0.001 | 0.56 | 0.7 |
| Validation VS MDDSB | 0.97 | < 0.001 | 0.97 | < 0.001 | 0.86 | 0.94 |

All abbreviations can be found in **STable 8**.

**STable 8.** List of Abbreviations Used in Figures and Tables.

| **Abbreviations** | **Full name** |
| --- | --- |
| n | number of subjects |
| n.a. | not available |
| SD | standard deviation |
| HC | healthy control |
| MDD | major depressive disorder |
| MDDNSI | major depressive disorder without suicidal ideation |
| MDDmSI | major depressive disorder with mild suicidal ideation |
| MDDmoSI | major depressive disorder with moderate suicidal ideation |
| MDDSSI | major depressive disorder with severe suicidal ideation |
| MDDSB | major depressive disorder with suicidal behavior |
| HAMD | Hamilton Depression Scale |
| AUD | auditory network |
| CON | cingulo-opercular network |
| DAN | dorsal attention network |
| DMN | default mode network |
| FPN | fronto-parietal network |
| SAN | salience network |
| SMN | sensory network |
| SUB | subcortical network |
| VAN | ventral attention network |
| VIS | visual network |
| FCI | Functional connectivity index |
| ROC | receiver operating characteristic |
| TPR | true positive rate |
| FPR | false positive rate |
| AUC | area under the curve |

**
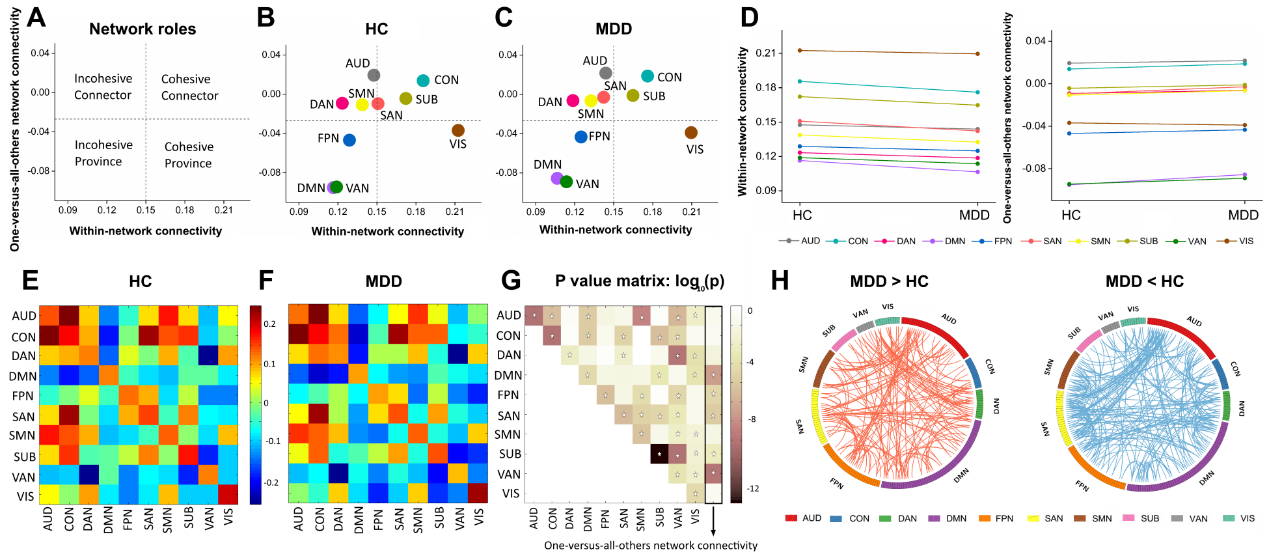
****SFig. 1.** Network Roles (**A**) in brain networks of HCs (**B**) and MDD patients (**C**). Line charts display the trajectory of within- and one-versus-all-others network connectivity between patients with MDD and HCs (**D**). Within- and pairwise between-network connectivity matrices of HCs (**E**) and MDD patients (**F**); P value matrix of group differences in within-, one-versus-all-others-, and pairwise between-network connectivity, the pentacle represents p value less than 0.05. (**G**). Circos plot represents significant group-level differences of neural connections among the ten RSNs between two groups using the network-based statistics method (**H**). All abbreviations can be found in **STable 8**.

**
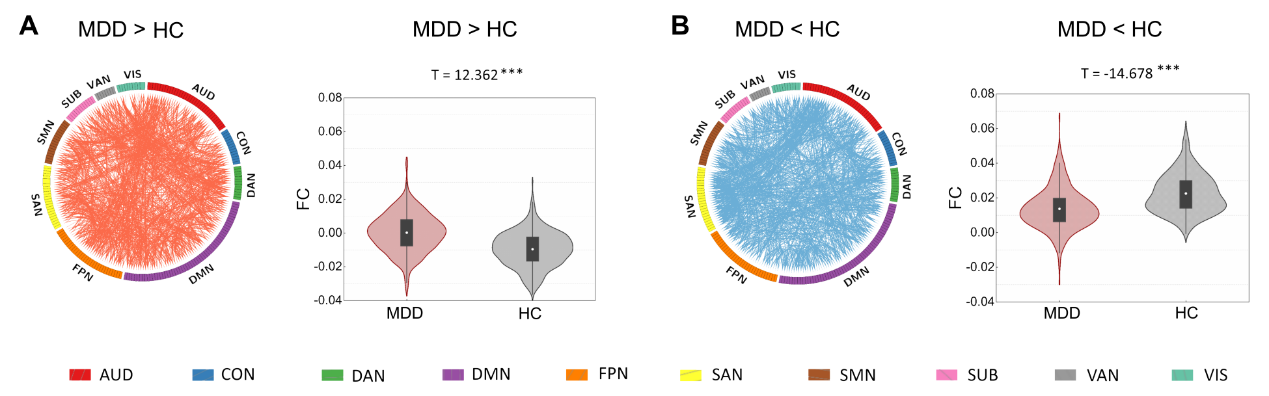
SFig. 2.** Differential Network Connections among the Ten RSNs in the MDD Patients Compared to Healthy Controls. **A** Group-level increased network connections among the ten RSNs in the MDD patients compared to HCs. **B** Group-level decreased network connections among the ten RSNs in the MDD patients compared to HCs. Each square color represents one of the ten networks. Red lines represent increased functional connectivity, blue lines represent decreased functional connectivity. The violin figures represent the group-level distribution of mean FC from the differential network connections among the ten RSNs in the MDD patient compared to HCs. All abbreviations can be found in **STable 8**.

**
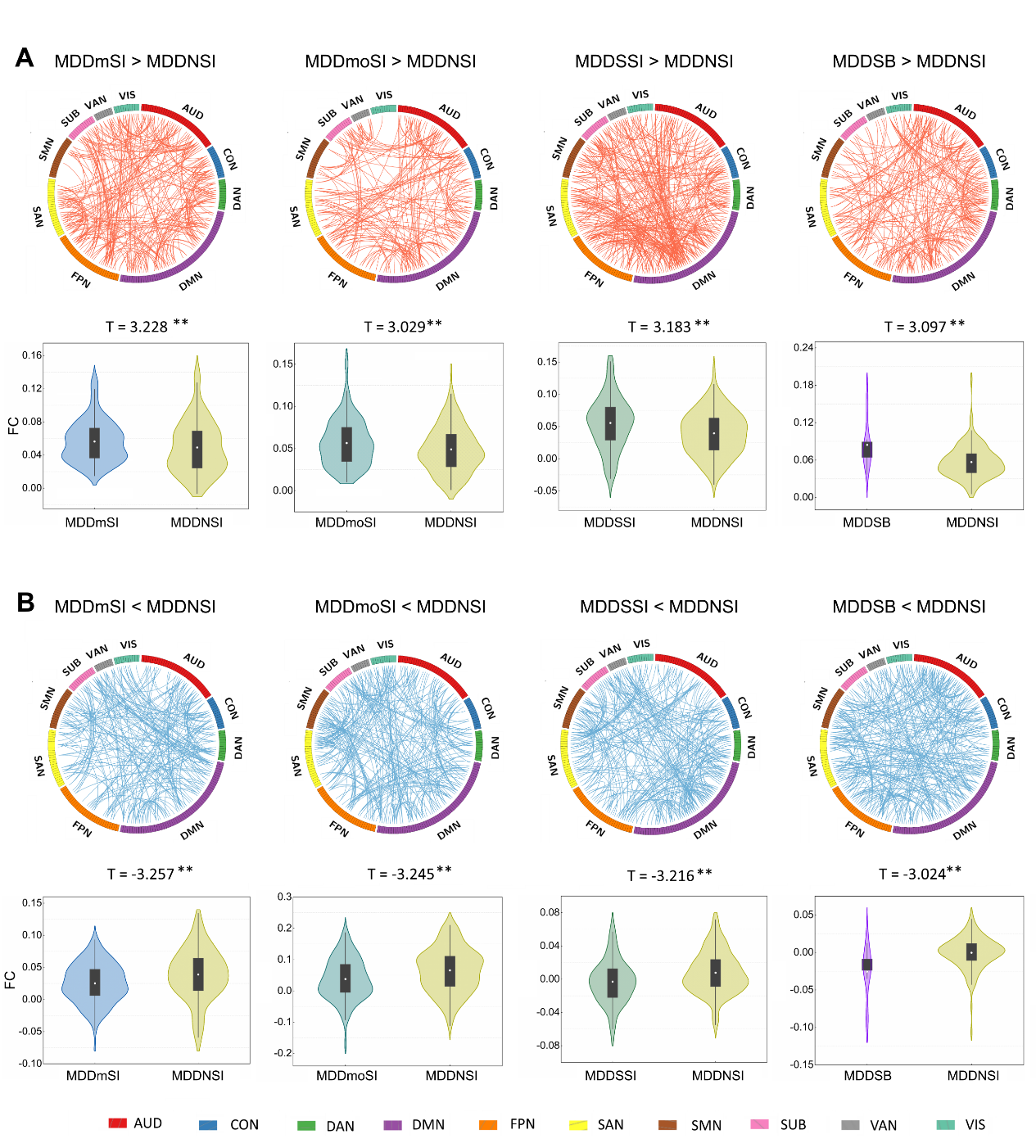
**

**Fig. S3.** Divergent Network Connections among the Ten RSNs in the Suicidality-Related MDD Patients Compared to MDD Patients without Suicidality. **A** Significantly increased network connections among the ten RSNs in MDD patients with suicidal ideation or behavior compared to MDDNSI. **B** Significantly decreased network connections among the ten RSNs in MDD patients with suicidal ideation or behavior compared to MDDNSI. Each square color represents one of the ten networks. Red lines represent increased functional connectivity, blue lines represent decreased functional connectivity. The violin figures represent the group-level distribution of mean FC from the differential network connections among the ten RSNs in the MDD patient with suicidal ideation or behavior compared to MDDNSI subjects. All abbreviations can be found in **STable 8.**

**
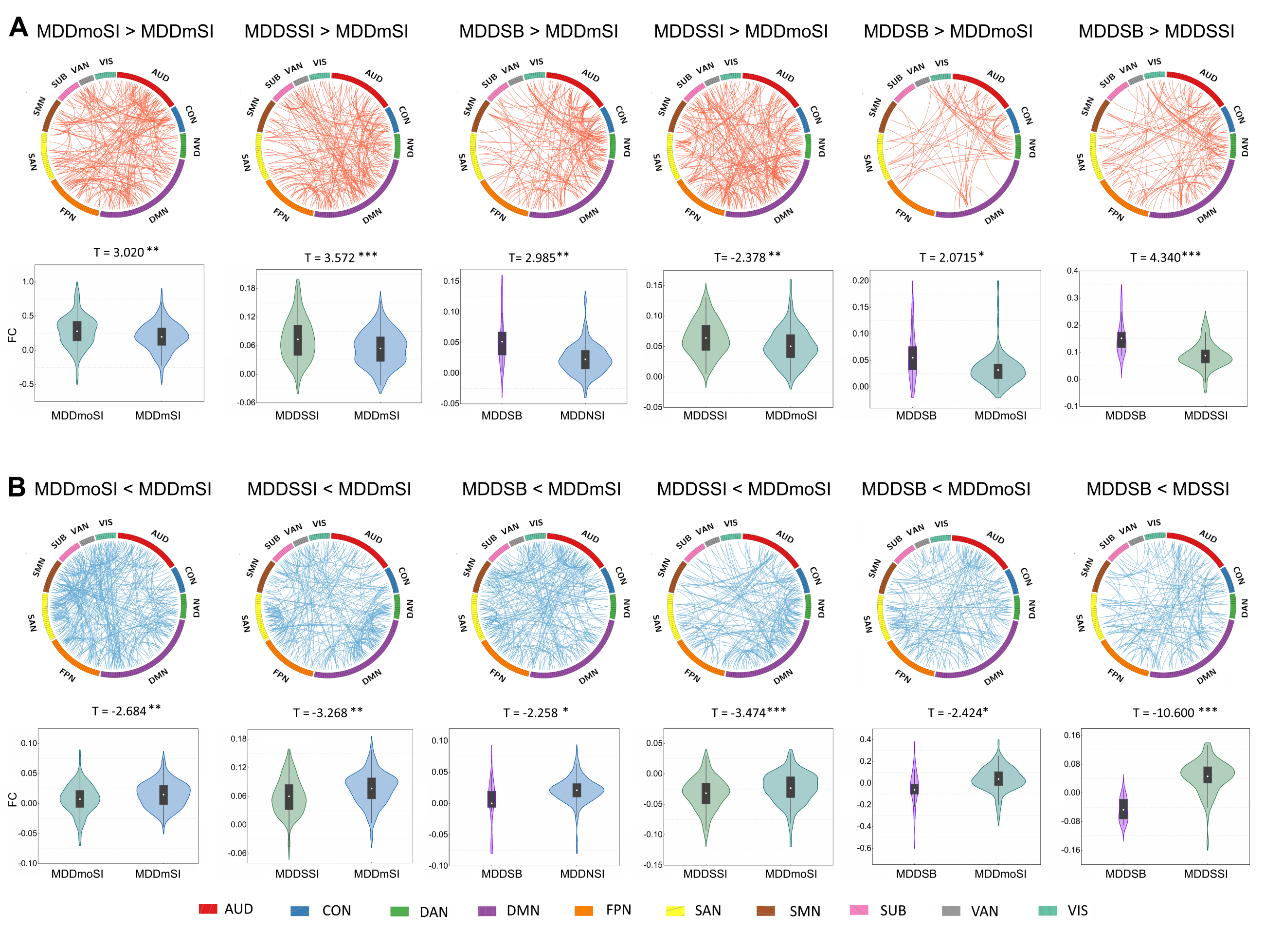
**

**SFig. 4.** Divergent Network Connections among the Ten RSNs within Suicidality-Related MDD Patients. **A** Group-level increased network connections among the ten RSNs in the four suicidality-related MDD patients. **B** Group-level decreased network connections among the ten RSNs in the four suicidality-related MDD patients. Each square color represents one of the ten networks. Red lines represent increased functional connectivity, blue lines represent decreased functional connectivity. The violin figures represent the group-level distribution of mean FC from the differential network connections among the ten RSNs in the suicidality-related MDD patients. All abbreviations can be found in **STable 8**.

**
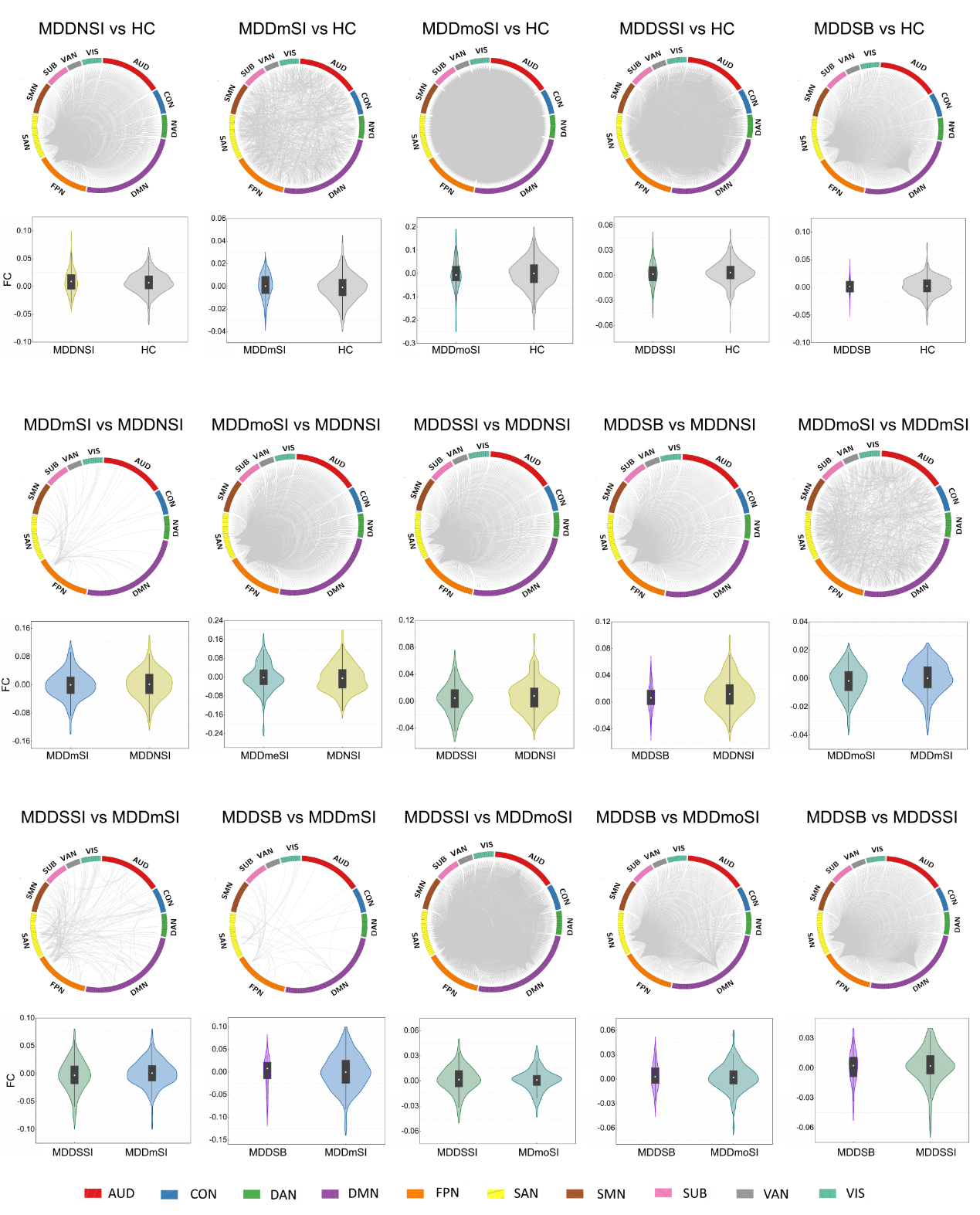
SFig. 5.** Common Network Connections of Group-Level Comparison among the Ten RSNs in the Five Sub-Groups of MDD Patients and Healthy Controls. Group-level common network connections among the ten RSNs identified by conjunction analysis between groups. Each square color represents one of the ten networks. Gray lines represent common functional connections. The violin figures represent the group-level distribution of mean FC from the common network connections among the ten RSNs in the MDD patient and HCs. All abbreviations can be found in **STable 8**.

**
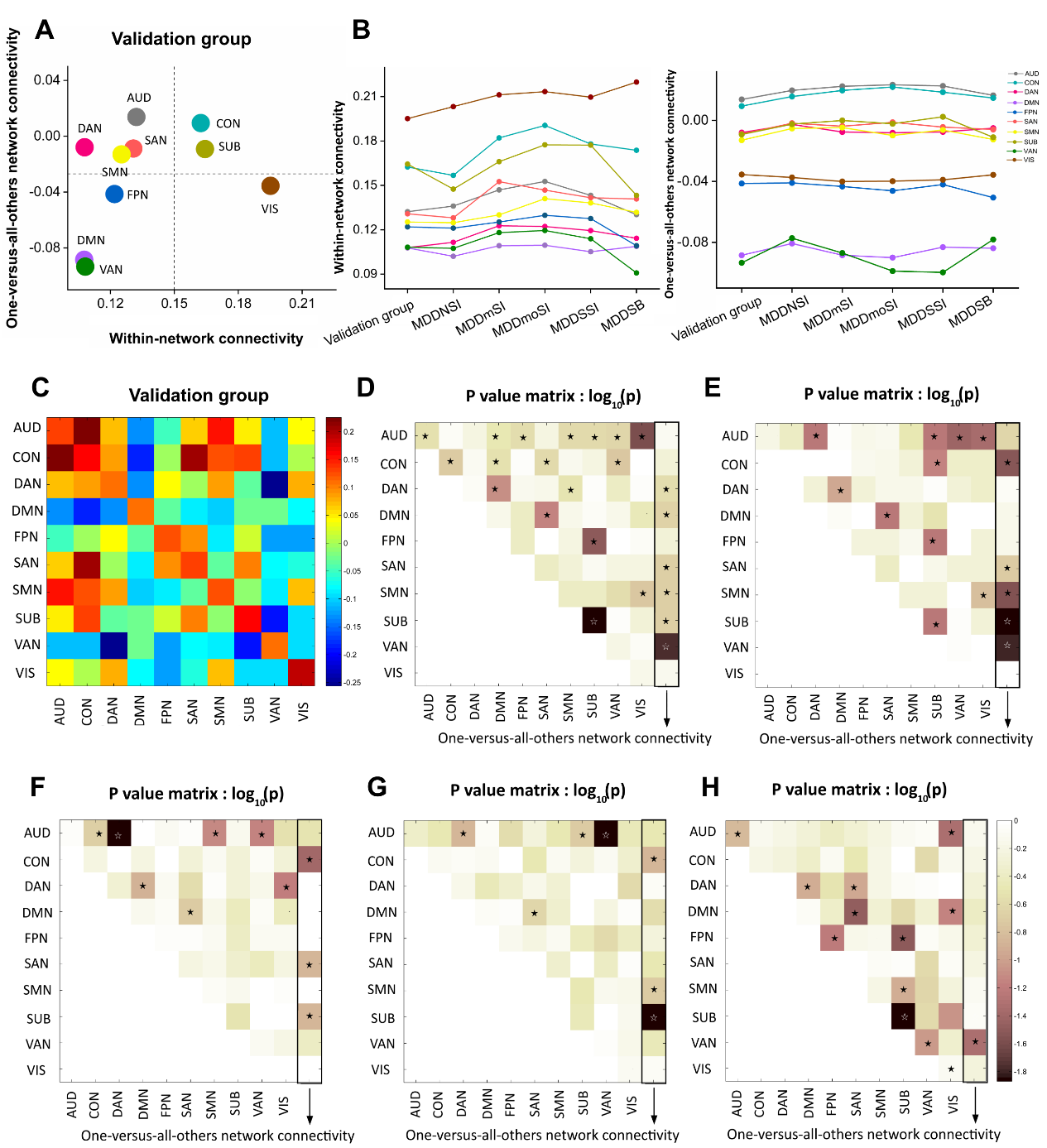
** **SFig. 6.** Network Roles of Validation Group. **A** Network roles in brain networks of validation group. **B** Line charts display the dynamic trajectory of within- and one-versus-all-others network connectivity among the five sub-groups of patients with MDD and validation group. **C** Within- and pairwise between-network connectivity matrices of validation group. **D-H** P value matrix of group differences in within-, one-versus-all-others-, and pairwise between-network connectivity (D: validation group vs MDDNSI; E: validation group vs MDDmSI; F: validation group vs MDDmoSI; G: validation group vs MDDSSI; H: validation group vs MDSB). All abbreviations can be found in **STable 8**.


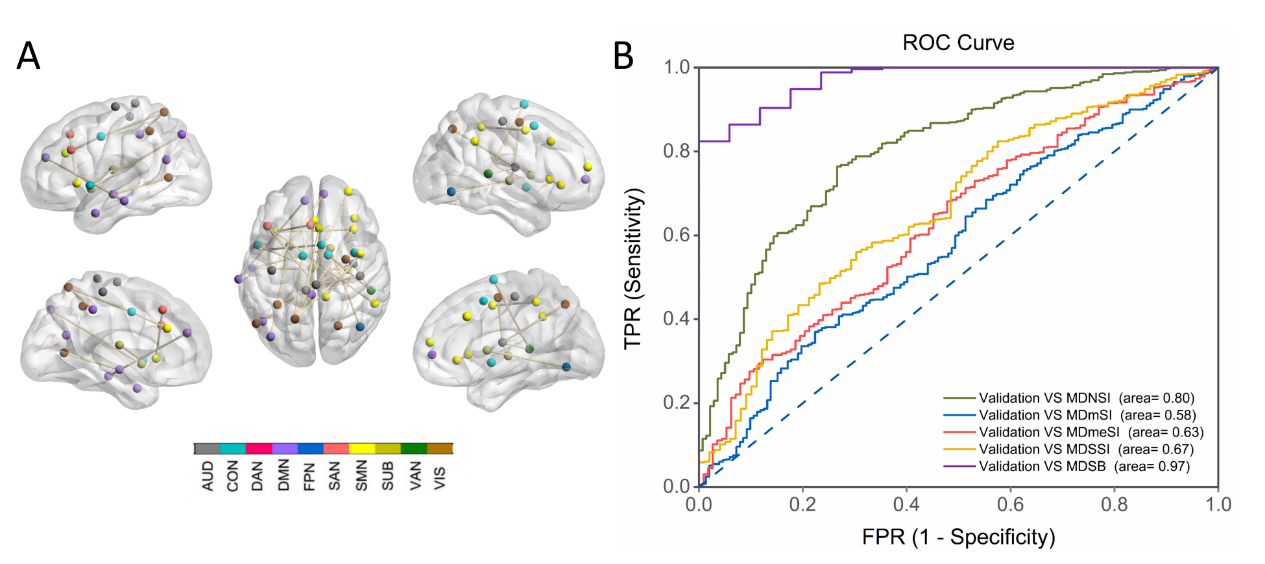
**SFig. 7.** Neuroimaging Biomarker for Classifying Validation Group and MDD Patients. **A** Thirty-one functional connections were used for classification between groups. Node colors represent Power-atlas cortical and subcortical regions consisting of 10 resting-state networks. **B** Functional connections that showed differences between all groups were used as the inputs for binary classification. All the p values of area under curve values were < 0.001. All abbreviations can be found in **STable 8**.
